# Detection of Plant Viruses and Disease Management: Relevance of Genetic Diversity and Evolution

**DOI:** 10.3389/fpls.2020.01092

**Published:** 2020-07-17

**Authors:** Luis Rubio, Luis Galipienso, Inmaculada Ferriol

**Affiliations:** ^1^ Centro de Protección Vegetal y Biotecnology, Instituto Valenciano de Investigaciones Agrarias, Moncada, Spain; ^2^ Plant Responses to Stress Programme, Centre for Research in Agricultural Genomics (CRAG-CSIC_UAB-UB) Cerdanyola del Vallès, Barcelona, Spain

**Keywords:** hybridization, PCR, loop-mediated isothermal amplification, next-generation sequencing, multiplexing, sensitivity, specificity, diagnosis

## Abstract

Plant viruses cause considerable economic losses and are a threat for sustainable agriculture. The frequent emergence of new viral diseases is mainly due to international trade, climate change, and the ability of viruses for rapid evolution. Disease control is based on two strategies: i) immunization (genetic resistance obtained by plant breeding, plant transformation, cross-protection, or others), and ii) prophylaxis to restrain virus dispersion (using quarantine, certification, removal of infected plants, control of natural vectors, or other procedures). Disease management relies strongly on a fast and accurate identification of the causal agent. For known viruses, diagnosis consists in assigning a virus infecting a plant sample to a group of viruses sharing common characteristics, which is usually referred to as species. However, the specificity of diagnosis can also reach higher taxonomic levels, as genus or family, or lower levels, as strain or variant. Diagnostic procedures must be optimized for accuracy by detecting the maximum number of members within the group (sensitivity as the true positive rate) and distinguishing them from outgroup viruses (specificity as the true negative rate). This requires information on the genetic relationships within-group and with members of other groups. The influence of the genetic diversity of virus populations in diagnosis and disease management is well documented, but information on how to integrate the genetic diversity in the detection methods is still scarce. Here we review the techniques used for plant virus diagnosis and disease control, including characteristics such as accuracy, detection level, multiplexing, quantification, portability, and designability. The effect of genetic diversity and evolution of plant viruses in the design and performance of some detection and disease control techniques are also discussed. High-throughput or next-generation sequencing provides broad-spectrum and accurate identification of viruses enabling multiplex detection, quantification, and the discovery of new viruses. Likely, this technique will be the future standard in diagnostics as its cost will be dropping and becoming more affordable.

## Introduction

Viral diseases are a major threat to sustainable and productive agriculture worldwide, resulting in losses of several billion dollars every year (Mumford et al., 2016). The highest impact occurs with emerging diseases, defined by a rapid increase in disease incidence, geographical range, and/or pathogenicity. The main factors driving virus emergence are: i) the agricultural systems based on monocrops with low genetic diversity and high plant density, which are more vulnerable to pathogens and pests; ii) world trade of plant material (germplasm and live plants) that moves viruses, hosts, and vectors to new regions and environments; iii) the climate change affecting the distribution area of hosts and vectors; and iv) the ability of viruses for rapid evolution and adaptation (Anderson et al., 2004; Jones, 2009; Elena et al., 2014).

Presently, curing plants once they have been infected by a virus is not feasible, unlike bacteria or fungi that can be treated with antibacterial or antifungal agents, respectively. So, disease management relies on preventing viruses from entering plants, or getting plants resistant to viral infection, using multiple strategies that must be developed specifically for each virus, host, and environment (pathosystem). Specific tools for virus diagnostics and identification are pivotal to set up and evaluate disease management. Here, the current state and progress of procedures used for virus detection are reviewed, discussing important features such as their sensitivity, specificity, versatility, portability, capacity for multiplexing, and virus quantification and designability. This review also includes basic concepts of genetic diversity and evolution of plant viruses and how they must be considered to improve detection. Finally, the main strategies for disease control are described, showing both the more suitable detection methods and how genetic diversity and evolution of virus populations can affect the efficiency and durability of some control strategies. This review follows a pragmatic approach aimed to guide plant pathologists to design and apply more accurate detection procedures for a more efficient management of viral diseases.

## Genetic Variability and Evolution of Plant Viruses

**Figure 1:**
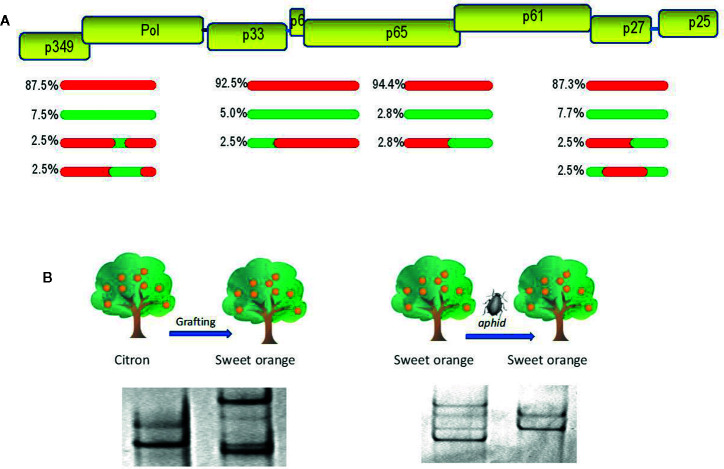
Evolutionary analysis of citrus tristeza virus (CTV). **(A)** A population of recombinants within the CTV isolate SY568-B6 (Vives et al., 2005). Above is a partial representation of the genomic map of CTV with boxes corresponding to genes. Red and green lines indicate sequence types with about 90% nucleotide identity between them. The relative frequency of each sequence type is indicated. **(B)** Genetic variation of the CTV isolate T317 after host change (Rubio et al., 2000) and the CTV isolate 408F after a transmission event by the aphid *Aphis gossypii* (d’Urso et al., 2000). Genetic variants are showed as electrophoretic bands after single-strand conformation polymorphism (SSCP) analysis.

Viruses have a great potential for high genetic variability due to their rapid replication and generation of large populations. Viruses with RNA genomes, comprising most plant viruses, and viroids have the highest mutation rate of any group of replicons, since RNA polymerases lack a proofreading activity (Domingo et al., 1996; Drake and Holland, 1999; Gago et al., 2009). The mutation rate is so high that replication from a single RNA molecule gives rise to a population of mutant sequences (haplotypes or variants) grouped around a master sequence, termed quasispecies (Holland et al., 1982; Moya et al., 2004). Populations of closely related viral or viroidal variants in individual plants have been reported (Ambrós et al., 1999; Kong et al., 2000; Gandía et al., 2005). Viral populations in individual plants can be even more complex, since mixed infections with different virus species (Juárez et al., 2013) or divergent variants of the same virus species (Rubio et al., 2001; Gómez et al., 2009) are frequent as a consequence of successive inoculations by vectors (e.g., insects). For example, a survey of seven tomato viruses in Sicily, Italy (Panno et al., 2012) showed that most plants (75.5%) presented multiple infections, whereas 17.8% were infected with a single virus, and only 6.7% were free of these viruses (**Table 1**). Synergistic interactions between different viruses and viroids in mixed infection can lead to increased virulence (symptoms and/or viral accumulation) or even new diseases (Wang et al., 2002; Wintermantel, 2005; Murphy and Bowen, 2006; Untiveros et al., 2007; Syller, 2012; Moreno and López-Moya, 2020). Mixed infections of two viruses also enable recombination, which, in addition to mutation, is another source of genetic variation and emergence of new viruses. Recombinants have been described between different species of plant viruses (Padidam et al., 1999; Chare and Holmes, 2006; Codoñer and Elena, 2008; Davino et al., 2012) or divergent viral strains (Rubio et al., 2013; Lian et al., 2013; Ferriol et al., 2014). Recombination seems a frequent event coupled to virus replication (Froissart et al., 2005; Sztuba-Solinska et al., 2011), so that populations of different recombinants have been found in individual plants (**Figure 1A**) (Vives et al., 2005; Weng et al., 2007). Recombination in RNA viruses is considered as a mechanism for rapid removal of many deleterious mutations produced during replication and regeneration of functional genomes (Moya et al., 2004).

**Table 1:** Multiple infections of viruses in tomato crops in Sicily, Italy.

N_v_ ^a^	N_m_ (%)^b^	PepMV ^c^	TSWV ^c^	ToTV ^c^	ToCV ^c^	CMV ^c^	ToMV ^c^	TICV ^c^	N_c_ (%)^d^
0	3 (6.7)	−	−	−	−	−	−	−	3 (6.7)
1	8 (17.8)	+	−	−	−	−	−	−	2 (4.4)
		−	+	−	−	−	−	−	2 (4.4)
		−	−	−	+	−	−	−	4 (8.9)
2	10 (22.2)	+	+	−	−	−	−	−	2 (4.4)
		+	−	−	+	−	−	−	2 (4.4)
		−	+	−	+	−	−	−	2 (4.4)
		−	+	−	−	+	−	−	1 (2.2)
		−	−	−	+	+	−	−	1 (2.2)
		−	−	−	+	−	−	+	2 (4.4)
3	13 (28.8)	+	+	−	+	−	−	−	2 (4.4)
		−	+	−	+	+	−	−	7 (15.6)
		−	+	−	+	−	+	−	1 (2.2)
		−	+	−	−	+	+	−	2 (4.4)
		−	−	−	+	+	+	−	1 (2.2)
4	8 (17.8)	+	+	-	−	+	+	−	1 (2.2)
		+	+	−	+	+	−	−	1 (2.2)
		−	+	−	+	−	+	+	2 (4.4)
		+	−	+	-	−	+	+	1 (2.2)
		+	−	−	+	+	-	+	1 (2.2)
		+	−	−	+	+	+	−	2 (4.4)
5	3 (6.7)	+	+	−	+	−	+	+	1 (2.2)
		+	+	−	+	+	+	−	1 (2.2)
		+	−	+	+	−	+	+	1 (2.2)
N_i_ (%)^e^		17 (37.8)	25 (55.6)	2 (4.4)	32 (71.1)	20 (44.4)	12 (26.7)	6 (13.3)	

Data obtained from **Table 2** in Panno et al., 2012.

a N_v_, number of viruses per plant.

b N_m_(%), number of plants and percentage (between parentheses) of uninfected (N_v_ = 0), or with single (N_v_ = 1), double (N_v_ = 2), triple (N_v_ = 3), quadruple (N_v_ = 4), or quintuple (N_v_ = 5) infections.

c Viruses: Pepino mosaic virus (PepMV), Tomato spotted wilt virus (TSWV), Tomato torrado virus (ToTV), Tomato chlorosis virus (ToCV), Cucumber mosaic virus (CMV), Tomato mosaic virus (ToMV) and Tomato infectious chlorosis virus (TICV). “+” indicates the presence and “−” the absence of a virus.

d N_C_ (%), number and percentage of plants infected by different virus combinations.

e N_i_ (%), number and percentage of plants infected by each virus.

The genetic variation produced by mutation and recombination is restricted and structured by the other three evolutionary forces: natural selection, genetic drift, and gene flow. Natural selection is a directional process by which the less fit virus variants will decrease their frequency in the population (negative or purifying selection) as a result of functional restrictions necessary for replication, movement between plant cells, transmission by vectors, and specific interactions between virus and host or virus and vector (Power, 2000; Schneider and Roossinck, 2001; Chare and Holmes, 2004). Positive or adaptive selection consists in the frequency increase of the fittest variants carrying genetic changes required to become adapted to new hosts and/or vectors (Agudelo-Romero et al., 2008; Ohshima et al., 2009; Peña et al., 2014). Genetic drift consists of stochastic changes in the frequencies of genomic variants in a finite population due to random sampling occurred during reproduction (Moya et al., 2004). The effect is a reduction of genetic variability and fixation of selectively neutral variants that is more evident after a rapid reduction of the population size by population bottlenecks or founder events, which can occur in different steps of the virus life cycle, such as virus movement between plant cells and transmission by vectors (Sacristán et al., 2003; Ali et al., 2006; Betancourt et al., 2008; Ali and Roossinck, 2010). **Figure 1B** shows genetic changes of citrus tristeza virus (CTV) isolates, revealed by single-strand conformation polymorphism analysis (explained below), after host change or vector transmission. Finally, gene flow (migration) among viral populations from distinct geographic areas is another factor shaping the genetic structure and variation, so that high migration rates favor genetic uniformity between populations decreasing the global genetic diversity (Moya et al., 2004). The rapid evolution of plant viruses implies that epidemiological and evolutionary processes interplay, and they must be considered together to understand and prevent viral emergence.

## Detection of Plant Viruses

### Serological and Molecular Techniques

In the last decades, rapid and specific serological (enzyme-linked immunosorbent assay, ELISA) and molecular techniques (molecular hybridization and DNA amplification) for the detection of plant viruses have been developed. ELISA is based on specific binding of viral proteins with antibodies (Clark and Adams, 1977), and molecular hybridization, on binding viral nucleic acids with sequence-specific DNA or RNA probes, due to their sequence complementarity (Hull and Al-Hakim, 1988). These binding events are visualized by attached markers based on fluorescent dyes, enzymes producing colorimetric or chemiluminescent reactions, radioactivity, or others.

Detection methods based on DNA amplification can be classified into two types: polymerase chain reaction (PCR) and isothermal amplification. PCR makes millions of DNA copies of a specific region of the viral genome that are usually visualized by electrophoresis or by hybridization with fluorescent probes. PCR can use as template genomic DNA, or complementary DNA obtained after reverse transcription (RT) of viral RNA. Amplification occurs in three steps: i) denaturation by heating at 90°C to 95°C to separate the double-stranded DNA (dsDNA) template into single strands; ii) annealing by cooling at 40°C to 60°C to allow the primers (two short DNA sequences of 15–40 nt) to bind the start and end of the target DNA; iii) extension by heating at 70°C to 75°C, in which a thermostable DNA polymerase synthesizes new DNA strands starting from the primers. These steps are repeated for 20 to 40 cycles, so the newly synthesized DNA segments serve as template in next cycles (Mullis and Faloona, 1987). The PCR product is visualized by electrophoresis, and it can be further characterized by Sanger sequencing (first-generation sequencing), enabling a more precise identification by comparison with known sequences from databases like GenBank (see below). Also, this approach is used to genotype virus populations, evaluate their genetic diversity, and study their evolution (see below). Real-time quantitative PCR (qPCR) is a variant of this technique that monitors the reaction progress by detecting a fluorescent reporter that binds to the dsDNA or is released from sequence-specific probes of 15 to 30 nt. This PCR variant can be used to quantify nucleic acids (see below).

Isothermal amplification can be achieved by different approaches (Olmos et al., 2007): i) Helicase dependent amplification (HAD) uses a helicase to separate the strands of dsDNA, allowing primer binding and extension by DNA polymerase at a constant temperature of about 65°C. ii) Recombinase polymerase amplification (RPA) uses a recombinase which forms a complex with primers to initiate amplification at a temperature between 37°C and 42°C. iii) Nucleic acid sequence-based amplification method (NASBA) uses a modified primer with the bacteriophage T7 promoter region that attaches to the RNA template. Reverse transcriptase and RNase H are used to synthesize complementary ds DNA, and a T7 RNA polymerase to synthesize complementary RNA strands resulting in amplification. iv) Loop-mediated isothermal amplification (LAMP) is based on auto cycling and high DNA strand displacement activity mediated by Bst polymerase from *Geobacillus stearothermophilus*, under isothermal conditions at 60°C to 65°C (Panno et al., 2020). Recently, new tools for molecular diagnosis have been developed based on prokaryotic clustered regularly interspaced short palindromic repeats (CRISPR) immunity system, widely applied for genome editing (Chertow, 2018).

### High-Throughput Sequencing

The advent of high-throughput sequencing (HTS) technologies, also known as next-generation sequencing, has led to a revolution in plant virus diagnosis (Maree et al., 2018; Villamor et al., 2019). HTS does not require any previous knowledge of viral sequences and can sequence millions or billions of DNA molecules in parallel, enabling the detection of all viruses present in a plant (virome), including those still unknown (Roossinck, 2015). HTS allowed elucidating the elusive etiology of some diseases (Vives et al., 2013; He et al., 2015), but often it is not possible to find a direct association between the disease and a particular virus (Tomašechová et al., 2020) among those detected in the infected plant. In this case, the diagnostic must be established by fulfilling Koch’s postulates, or at least by finding a tight association between the disease and the presence of a certain virus in field surveys (Mumo et al., 2020).

HTS can be divided into two types. Second-generation sequencing is based on the preparation of random libraries of DNA fragments when DNA is used as starting material, or of cDNA obtained by retrotranscription of the RNA with random primers or oligodT. These libraries are clonally amplified, bond to synthetic DNA adapters and sequenced in parallel. This produces a large number of short sequence reads (100–500 nt) that are assembled by connecting overlapping sequence reads according to nucleotide identity by informatic analysis, e.g., Geneious package (www.geneious.com). Several platforms for second-generation sequencing have been developed by different companies such as Roche 454, Illumina, Solid and Ion Torrent (Bleidorn, 2016; Goodwin et al., 2016; Heather and Chain, 2016; Villamor et al., 2019). In addition to detection of new plant viruses and viroids, HTS is being used for studies on epidemiology, synergistic interactions between viruses, and genetic diversity and evolutionary mechanisms of virus populations (Kehoe et al., 2014; Hadidi et al., 2016; Pecman et al., 2017; Roossinck, 2017; Tan et al., 2019; Xu et al., 2019).

Third-generation sequencing is based on sequencing single molecules in real-time without the need for clonal amplification, thus shortening DNA preparation time and giving long reads of several kilobases (Goodwin et al., 2016; van Dijk et al., 2018). Long reads are more appropriate for genome sequencing, genotyping, and detecting recombination. However, third-generation sequencing needs further improvement since error rates are still much higher than in second-generation sequencing. Several techniques are being developed by different companies such as single-molecule real-time (SMRT) and nanopore sequencing. SMRT sequencing uses a flow cell with millions of individual picolitre wells with transparent bottoms (zero-mode waveguides) with a DNA polymerase fixed. Incorporation on each single-molecule template per well is continuously visualized with a laser and camera system that records the color and duration of emitted light, as the labeled nucleotide momentarily pauses during incorporation at the bottom of the wells. Nanopore sequencing is based on translocating the DNA or RNA through a nanopore (in membrane proteins or synthetic materials such as silicon nitride and aluminum oxide), where an ionic current pass by setting a voltage. The DNA passing through the nanopore changes the current depending on the shape, size and length of the DNA sequence. Nanopore sequencing has several advantages such as the relatively low cost compared to other HTS technologies, high mobility due to the sequencer small size and rapid sample processing, without the need for reverse transcription for RNA viruses (Kiselev et al., 2020). This technology has been recently used to detect some plant viruses, such as plum pox virus (PPV) and tomato yellow leaf curl virus (TYLCV), and discover new plant viruses (Bronzato Badial et al., 2018; Chalupowicz et al., 2019; Naito et al., 2019).

### Accuracy of Virus Diagnosis

Detection procedures must be optimized for accuracy, measured as sensitivity and specificity, which are the statistical measures of performance of binary classification tests (Sharma et al., 2009). Sensitivity measures the proportion of actual positives which are classified as such (probability of true positives) and specificity measures the proportion of negatives which are correctly identified (probability of true negatives). Other measures of accuracy are positive predictive value, defined as the proportion of positive samples correctly diagnosed, and negative predictive value, or proportion of samples with negative results correctly diagnosed (**Table 2**). However, the predictive values depend on the infection prevalence in the samples tested and do not apply universally (Olmos et al., 2007).

**Table 2:** Measure of accuracy in diagnostic tests.

	Virus present	Virus absent	
Test positive	a (True positives)	b (False positives) Type I error	→ Positive predictive value a/(a+b)
Test negative	c (False negatives) Type II error	d (True negatives)	→ Negative predictive value c/(c+d)
	↓ SENSITIVITY a/(a+c)	↓ SPECIFICITY b/(b+d)	

*a, b, c, and d represent number of samples (plants).

Low virus titer can limit sensitivity, producing false negatives when the virus concentration is under the technique detection threshold. Usually, the molecular techniques are more sensitive than the serological ones. Conventional PCR is much more sensitive than molecular hybridization. Some modalities of PCR are even more sensitive, such as qPCR and nested PCR (this uses two successive runs of PCR with a second primer pair to amplify a secondary target within the product of the first run). LAMP exhibits a sensitivity in the order of qPCR and is less affected than PCR by inhibitors (phenols, tannins, and complex polysaccharides), which are often a cause of false negatives. Paradoxically, the high sensitivity of the amplification techniques can be a problem, as contamination of reagents and instruments with amplicons from previous samples and cross-contamination between samples can produce false positives reducing specificity.

An important factor affecting the accuracy of the serological and molecular detection methods is the genetic variability within each virus species and the genetic relationships with other virus species. Since these methods are based on specific binding (protein with antibody or nucleic acids with probes or primers), some dissimilar virus variants can fail to react giving false negatives. For example, universal detection of PPV by ELISA with monoclonal antibodies failed for some PPV isolates (Sheveleva et al., 2018). On the other hand, false positives by cross-reactions of antibodies with related viruses have been described for some viruses, e.g., arabis mosaic virus (ArMV) (Frison and Stace-Smith, 1992). Unlike antibody production, primers and probes are better suited to be optimized by considering the genetic variability. However, genetic variation of viruses is often neglected, and accuracy is tested just with samples from local surveys harboring genetically similar virus isolates. Thus, some detection protocols can fail when applied with the same reagents (probes or primers) in other geographical areas or after the emergence of divergent variants, e.g., pepino mosaic virus (PepMV) and apple chlorotic leafspot virus (ACLSV) (Mansilla et al., 2003; Spiegel et al., 2006).

**Figure 2:**
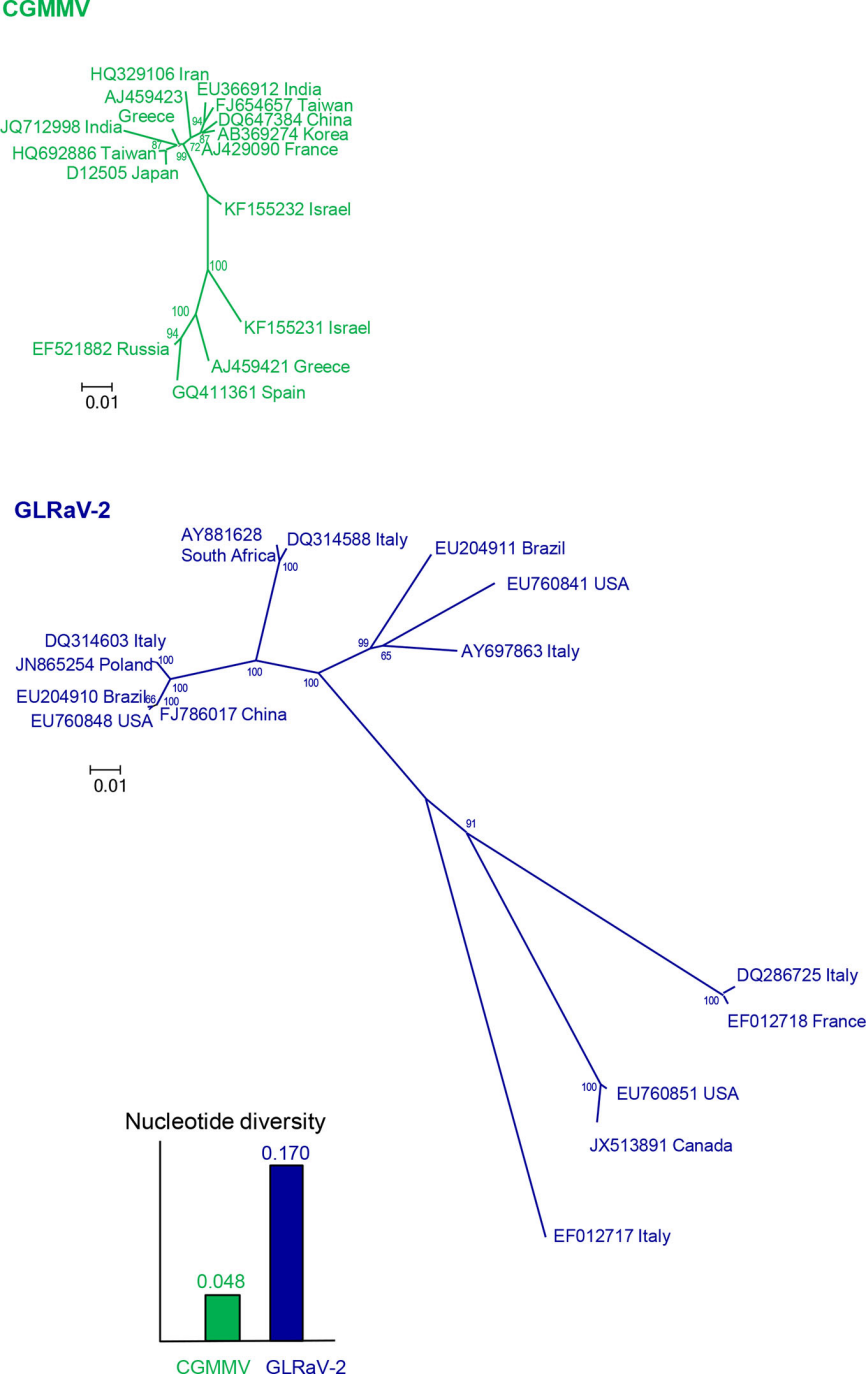
Nucleotide diversities and unrooted neighbor-joining trees of the coat protein gene of 15 isolates of cucumber green mottle mosaic virus (CGMMV) and grapevine leafroll-associated virus 2 (GLRaV-2). Branch lengths are proportional to the genetic distances and bootstrap values ≥ 65% are indicated.

To design accurate probes or primers for a given virus, the first step is to get a picture of the genetic variation and structure by gathering as many nucleotide sequences as possible from isolates of that virus and from genetically related viruses. Sequences of specific genomic regions or complete genomes can be determined from purified or cloned PCR products or by HTS from viral samples and retrieved from databases like GenBank (https://www.ncbi.nlm.nih.gov/). The genetic diversity and structure can be estimated easily with the MEGA X software (Kumar et al., 2018), after alignment with the algorithm CLUSTALW (Higgins et al., 1994) implemented in MEGA. Nucleotide diversity is the mean distance (proportion of nucleotide differences) between sequence pairs and can be considered as a measure of the genetic variation within a virus population. In this case, p-distance should be used instead of nucleotide substitution models as this analysis is aimed to know the actual genetic differences for application in diagnostic and not the evolutionary changes that occurred. The genetic structure can be visualized with phylogenetic trees, which can be inferred with different methods, such as Neighbor-Joining, Maximum Likelihood and Maximum Parsimony. As an illustration, **Figure 2** shows the nucleotide diversity and the phylogenetic relationships of randomly selected worldwide isolates of cucumber green mottle mosaic virus (CGMMV) and grapevine leafroll-associated virus 2 (GLRaV-2). The nucleotide diversity of GLRaV-2 is higher, so it is more challenging to develop an accurate detection method for GLRaV-2 than for CGMMV. Since the viral population of GLRaV-2 is structured in eight groups or clades, at least one isolate per clade should be considered to develop detection and disease control procedures for this virus.

For universal detection of a virus by PCR, primers should be designed from short sequence stretches with conserved nucleotide positions among all available sequences and they should be degenerated to cover possible genetic variation not found in the sequences analyzed (explained in *Detection Levels*). For example, detection of PepMV by RT-PCR (Mansilla et al., 2003) failed for new PepMV isolates and universal detection was only achieved after designing primers targeting conserved sequence stretches among PepMV isolates (Ling et al., 2007). For molecular hybridization, different genomic regions should be considered for probe design since the genetic diversity can vary widely along the genome due to different selective pressures or recombination. For example, broad bean wilt virus 1 (BBWV-1), with a bipartite single-stranded RNA (ssRNA) genome, showed the lowest nucleotide diversity in the 5′ terminal sequence of RNA 1 (Ferriol et al., 2014). Thus, only a DNA probe binding this genomic region allowed universal detection of BBWV-1 by molecular hybridization (Ferrer et al., 2008).

HTS enables accurate and unbiased identification of viruses unlike the other techniques (ELISA, molecular hybridization or amplification) requiring a specific binding that can fail to detect some genetic variants. HTS generates hundreds of megabases to gigabases of nucleotide sequence reads in a single run providing a good sensitivity (Santala and Valkonen, 2018). However, cross-contamination can produce false positives, so it is necessary validation with other techniques, such as PCR (Maree et al., 2018).

### Detection Levels

Detection and identification of viruses are based on assigning a virus from a plant sample to a group of viruses sharing common characteristics. In most cases, the level of detection is the virus species, but it can also be set for higher taxonomic units such as genus or family, or lower units like strain (virus variants with distinctive biological or molecular characteristics).

Serological techniques usually detect viruses to the species level and, in some cases, they allow discrimination between virus strains (serotypes) using monoclonal antibodies (Permar et al., 1990; Myrta et al., 2000; Sheveleva et al., 2018). Molecular hybridization has been used mostly to detect virus species (**Supplementary Table S1**), but the detection level can be modified to a certain extent by using different probes and hybridization conditions. The stability of the hybrid complexes depends on the probe length and G-C content, the probe type (DNA or RNA), and the number of global or local mismatches (nucleotide distance) between target and probe. Therefore, the distribution of nucleotide variation along the virus genome should be considered to modulate the level of detection and to test for accuracy. Regarding the hybridization conditions, a more selective detection can be attained by using more stringent conditions (higher incubation temperature, lower salt concentration or adding denaturing agents like formamide), that reduce the number of mismatches permitted to occur. Thus, probes from variable genomic regions with stringent conditions have been used to discriminate between virus strains or isolates (Narváez et al., 2000; Ferrer et al., 2008). Designing probes complementary to regions conserved within taxonomic units higher than species is challenging given the high nucleotide variation among species. To our knowledge, only two cases have been reported: i) A single RNA probe derived from the 5′ untranslated of BBWV-1 was able to hybridize with other members of the genus *Fabavirus* (Ferriol et al., 2015). This genomic region contains several perfect or near-perfect repeats of ten nucleotides that allow hybridization despite the low nucleotide identity between these virus species. ii) A polyprobe with seven conserved motifs of the genus *Potyvirus* allowed detection of 32 viruses of this genus by hybridizing at low stringency conditions (Sánchez-Navarro et al., 2018).

PCR techniques are the most versatile and primers have been designed for different detection levels from families and genera (**Supplementary Table S2**) to strains and genetic variants (Ruiz-Ruiz et al., 2009b; Debreczeni et al., 2011), whereas isothermal amplification has been limited to the species level (**Supplementary Table S3**). Obtaining primers specific for genera or families is more challenging than for virus species given the increasing nucleotide diversity of higher taxons. Primer design requires searching for conserved nucleotide positions among the members of the genus or family, which usually correspond to sequence motifs with relevant biological functions, and therefore, subjected to strong negative selection. The conserved positions can occur at the nucleotide level due to structural constraints, codon usage, or at sites where regulatory proteins bind (Koonin, 1991; Adams and Antoniw, 2004; Watters et al., 2017), but most are at amino acid level. For example, primers for the subfamily *Comovirinae* (composed of the genera *Comovirus*, *Fabavirus* and *Nepovirus*) were designed based on amino acid motifs of the RNA-dependent RNA polymerase: (T/V)YGDDN(V/L) and TSEG(Y/F)P (Koonin, 1991; Maliogka et al., 2004).

**Figure 3:**
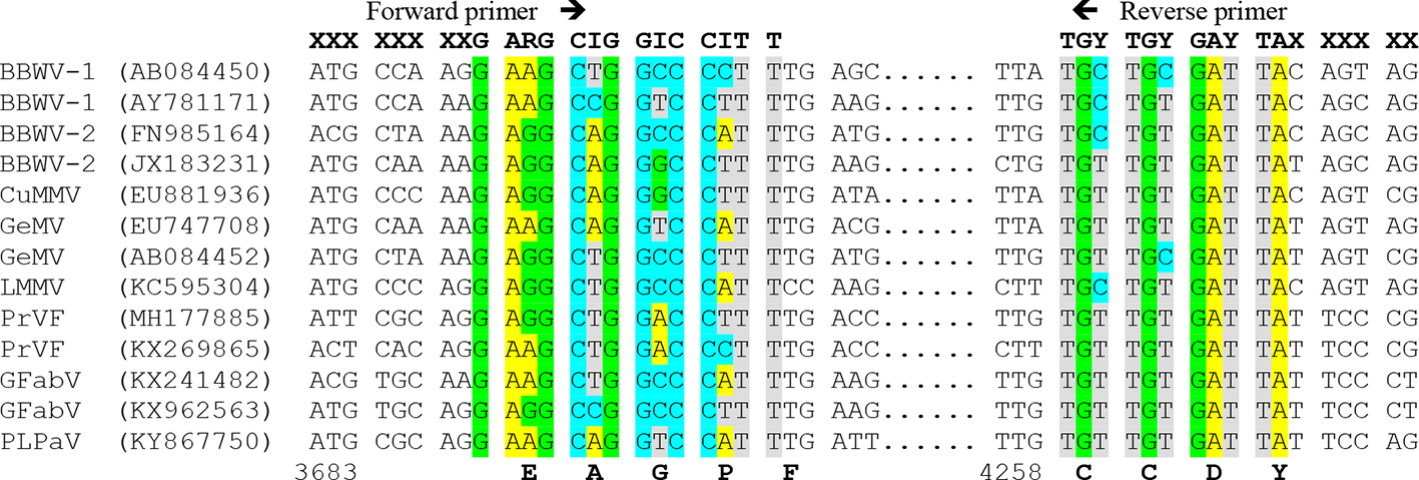
Multiple nucleotide alignment of RNA 1 of different members of the genus *Fabavirus*, showing a hypothetical design of degenerated primers based on conserved nucleotide positions. On top are hypothetical primers with degenerate sites: R=A+G, Y= C+T and I (inosine)= A+C+G+T, and X= less restricted nucleotides. Virus species of the genus *Fabavirus* are *Broad bean wilt virus* 1 (BBWV-1), BBWV-2, *Cucurbit mild mosaic virus* (CuMMV), *Gentian mosaic virus* (GeMV), *Lamium mild mosaic virus* (LMMV), *Prunus virus* F (PrVF), *Grapevine fabavirus* (GFabV) and the tentative member peach leaf pitting-associated virus (PLPaV). GenBank accession numbers are between parentheses. Below are the nucleotide positions for GenBank accession AY781171 and the conserved amino acids.

To design primers detecting the members of a genus or family, at least one sequence per each virus species should be used for alignment, preferably a codon-based or amino acid alignment. Since the genetic code is redundant, the primer must be degenerate, that is, composed of a mixture of almost identical primers differing in some positions and covering all possible nucleotide combinations for that protein sequence. The degeneracy level should be reduced as much as possible due to its negative effect in sensitivity (only a small proportion of the primers would bind the target) and specificity (some primers can bind to nontarget sequences). Several approaches can be used, such as i) limiting the degenerate sites to the last 9 to 12 nucleotides from the 3′ terminus, which are critical for PCR amplification, whereas some mispairings at the 5′ terminus are allowed; ii) choosing low degeneracy (one- or two-fold) codons, particularly at the 3′ terminus; and iii) using modified nucleotides such as inosine (I) for four-fold degenerate sites that can base-pair with the four normal nucleotides: A, C, G and T. As an illustration, **Figure 3** shows conserved amino acid positions in the genus *Fabavirus*, which can be used for a hypothetical universal detection of viruses within this genus. The PCR products obtained with conserved primers for a genus or family can be further purified and sequenced to identify the viral species or discover new ones (Zheng et al., 2010). In plants infected with two or more species of the same genus or family, it is necessary to clone the PCR products and sequence individual clones to identify each virus species. In some cases, the PCR products obtained with conserved primers are of distinct size for each virus species and can be easily discriminated by electrophoresis (James and Upton, 1999; Ferrer et al., 2007).

Tools to detect small genetic variations are also necessary given the great potential of viruses to generate high genetic and biological variation (genetic variants can display different properties, such as host range, virulence and resistance-breakdown). Several techniques based on PCR or using PCR products as templates have been used for genotyping: i) Randomly amplified polymorphic DNA (RAPD)-PCR uses a single short primer with an arbitrary nucleotide sequence (8–12 nucleotides) to produce different random segments depending on the target amplified, which are visualized by electrophoresis. This technique does not require knowledge of the target DNA sequence, but its reproducibility is low and has been applied only for few plant viruses (Williams et al., 1990; de Araújo et al., 2007); ii) Restriction fragment length polymorphism (RFLP) analysis is based on digestion of the PCR products with restriction enzymes and electrophoretic separation of the resulting restriction fragments according to their length, revealing sequence differences within the restriction sites. This technique has been used to differentiate isolates of some plant viruses, such as prunus necrotic ringspot virus (PNRSV), TYLCV and CTV (Gillings et al., 1993; Hammond et al., 1998; Font et al., 2007); iii) Single-strand conformation polymorphism (SSCP) analysis is based on electrophoresis of denatured dsDNA in non-denaturing gels so migration of single-stranded DNA depends on its conformation determined by its nucleotide sequence and the electrophoretic conditions. This technique is very resolutive and can detect small differences, even of a single nucleotide, but it is very sensitive to minute changes in the electrophoretic conditions hindering reproducibility. The main advantage of SSCP analysis is the ability to detect different genetic variants (visualized as electrophoretic bands) within a sample (plant), allowing to assess within-isolate genetic variation rapidly. This technique followed by sequencing of the different haplotypes detected has been used to evaluate the genetic variation of some plant viruses, such as cucumber mosaic virus (CMV), citrus psorosis virus (CPsV) and CTV (Rubio et al., 1996; Rubio et al., 1999; Vives et al., 2002; Lin et al., 2003; Martín et al., 2006). iv) Real-time qPCR has been used to differentiate virus strains by high resolution melting DNA curve analysis with SYBR Green or by using TaqMan™ fluorescent probes specific for each strain (Varga and James, 2005; Ruiz-Ruiz et al., 2007; Bester et al., 2012).

HTS techniques are the most powerful and versatile since the nucleotide sequences can be used not only to estimate the genetic variation and structure of virus populations but also to identify and ascribe a virus sample to different taxonomic levels or discover new virus species, genera or families (Kreuze et al., 2009; Wu et al., 2015; Pecman et al., 2017; Verdin et al., 2017), according to its nucleotide or amino acid identity with known sequences in databases (GenBank) or the presence of sequence motifs. This task can be easily carried out with the algorithm BLAST (https://www.ncbi.nlm.nih.gov/BLAST/), which compares the query sequence with all sequences from databases and find those that are more similar.

Recombination can produce biological and genomic variants of a virus that can be very similar in one genome region and very divergent in other. Thus, the complete genome or different regions of it should be analyzed for detection and identification of recombinant variants. Recombination can be detected by comparing nucleotide identity and/or phylogenetic relationships along the genome, which can be performed with different procedures implemented in the package RDP 4 (Martin et al., 2015). For example, identification of CTV strains requires different sets of primers (Roy et al., 2010) as recombination has played an important role in shaping CTV genome (Martín et al., 2009). HTS can be useful to detect recombination since full-length or almost full-length viral genomes are sequenced rapidly, in a single analysis (Akinyemi et al., 2016; Silva et al., 2019), unlike genome walking that requires successive steps of PCR, Sanger sequencing and primer design. Third-generation sequencing of single molecules seems more appropriate to identify recombination (Viehweger et al., 2019) than second-generation sequencing methods that can produce recombinant artifacts, as genomic sequences are assembled from short sequences. Nevertheless, it is convenient to confirm the recombinants by PCR followed by Sanger sequencing.

### Multiplexing

Procedures to detect and identify various viruses or virus strains in a single assay simultaneously reduce time and cost of the analysis (see Pallás et al., 2018 for a comprehensive review), and are especially suitable for evaluating mixed infections in individual plants. The detection of individual viruses in a sample is mainly based on three approaches: i) spatial separation of detection sites (wells or spots); ii) separation of distinctly sized amplicons by electrophoresis; and iii) using a different label for each virus (Dincer et al., 2017).

Multiplex PCR or RT-PCR is the amplification of multiple targets simultaneously in a single PCR by using several primer pairs specific for each target. Development of a multiplex PCR or RT-PCR assay is often complex since primers must comply with several conditions: i) similar melting temperatures (similar length and G-C content) so that all primers can function under the same PCR conditions; ii) compatibility, avoiding cross-binding and competition; iii) similar sensitivity; and iv) flanking genomic regions of different sizes so that the amplicons of each target can be separated and visualized by gel electrophoresis. The last constriction can be avoided by using primers labeled with different color fluorescent dyes or coupling the PCR with hybridization with specific probes (James et al., 2006). Multiplex PCR or RT-PCR have been used to identify: i) the main viruses infecting a particular crop, such as tomato, tobacco, legumes, potato, ornamentals, cucumber and olive (Bariana et al., 1994; Bertolini et al., 2001; Dai et al., 2012; Panno et al., 2012; Ge et al., 2013; Ali et al., 2014; Pallás et al., 2018); ii) viruses from the same genus (Uga and Tsuda, 2005; Hu et al., 2010; Panno et al., 2014); and iii) different strains of a viral species (Hammond et al., 1999; Nie and Singh, 2003; Huang et al., 2004; Alfaro-Fernández et al., 2009; Bester et al., 2012). Multiplex real-time qPCR with Taqman probes labeled with different fluorescent dyes have been used to identify viruses from the same crop (Abrahamian et al., 2013; López-Fabuel et al., 2013; Malandraki et al., 2017), and strains or isolates from the same viral species (Varga and James, 2005; Debreczeni et al., 2011). The main problem of multiplex PCR is that only a limited number of targets can be amplified simultaneously since the more primers are used, the higher is the probability of incompatibility between some of them. Also, there is a limitation in the number of products of different sizes that can be resolved by electrophoresis or the number of fluorescent dyes that can be used (Boonham et al., 2007). Multiplex LAMP has also been developed for the simultaneous detection of some plant viruses (Zhang J. et al., 2018; Wilisiani et al., 2019). Molecular hybridization with cocktails of probes or polyprobes (probes linked in tandem) has been used to detect different viruses affecting a crop (Sánchez-Navarro et al., 1999; Saade et al., 2000; Herranz et al., 2005; Aparicio et al., 2009; Minutillo et al., 2012), although further analyses are necessary to identify each virus.

Microarray analyses can screen many samples simultaneously. They can be based on serology, but most are based on molecular hybridization (Boonham et al., 2007; Boonham et al., 2014; Charlermroj et al., 2014). Capture probes corresponding to different viruses and/or genomic regions are attached to a solid support (usually glass) and the sample to be examined is fluorescently labeled so the identity of the virus or viruses present in the sample is determined by the fluorescent positions on the array. Capture probes can be produced from PCR products (200–1000 bp in length) or synthetic oligonucleotides (20–70 nucleotides in length). Probe design must consider probe length, melting temperature, GC content, secondary structure caused by self-annealing and hybridization free energy since they affect sensitivity and specificity. Short oligonucleotides (15–25 bases) are better to discriminate small sequence differences but exhibit reduced sensitivity. Oligoprobes can be designed for different detection levels (genus, species and strain) by choosing conserved or variable sequence stretches. Sensitivity and specificity can be improved controlling the hybridization temperature and buffer composition (Boonham et al., 2007). Microarrays have been used to detect: i) viruses infecting a particular crop, such as tomato, cucurbits, potato and grapevine (Bystricka et al., 2003; Engel et al., 2010; Sip et al., 2010; Tiberini et al., 2010; Tiberini and Barba, 2012); ii) different viral species of a genus (Wei et al., 2009); and iii) isolates or variants of the same virus (Deyong et al., 2005; Pasquini et al., 2008). Microarrays have been improved to detect hundreds of plant viruses, including genus-specific oligoprobes (Zhang et al., 2010; Nicolaisen, 2011; Nam et al., 2014).

Microsphere technology, like Luminex xMAP, can detect up to 500 targets in a single sample. It is based on microspheres (beads) coated with specific antibodies or oligonucleotides, which capture respectively viruses or PCR products obtained from their genetic material. The beads have been dyed into spectrally distinct sets, or “regions,” allowing them to be individually identified. After binding, the target is labeled by specific conjugated antibodies or probes which will give a fluorescent signal. The color code of the bead in combination with the fluorescent signal identifies a unique combination (Boonham et al., 2014). Luminex xMAP system has been used to detect viruses infecting a crop (Lim et al., 2016), viruses belonging to a genus (van Brunschot et al., 2014; Bald-Blume et al., 2017a), and strains or variants within a virus species (Bald-Blume et al., 2017b).

HTS is the most powerful technique for multiplex detection as it can identify and discover an unlimited number of viruses and virus variants within a plant (Jones et al., 2017).

### Quantification

Estimating the amount of a specific virus provides more precise information than just determining the presence or absence of that virus. ELISA and molecular hybridization can be used for rough quantification of viral particles or nucleic acids based on the signal intensity (Rubio et al., 2003a; Rohrman et al., 2012). Real-time qPCR is a very accurate procedure to estimate virus titer with a wide dynamic range and great sensitivity. The principle is to monitor in each cycle the increase of fluorescence. The cycle at which amplification is observed (cycle threshold, CT) is related to the inverse Log of the quantity of target being amplified (Boonham et al., 2014). Real-time qPCR or RT-qPCR has been developed for several plant viruses (Mumford et al., 2000; Picó et al., 2005; López et al., 2006; Ling et al., 2007; Hongyun et al., 2008; Ruiz-Ruiz et al., 2009a; Debreczeni et al., 2011; Ferriol et al., 2011; Sharma and Dasgupta, 2012; MacKenzie et al., 2015; Herrera-Vásquez et al., 2015). It has been applied to evaluate some disease control methods such as i) study interactions between viruses in mixed infections (Mortimer-Jones et al., 2009; Abrahamian et al., 2013), which can be used for control based on cross protection (Ruiz-Ruiz et al., 2009b; Hanssen et al., 2010); ii) estimation of correlation between virus accumulation and transmission by insect vectors (Olmos et al., 2005; Rotenberg et al., 2009; Ferriol et al., 2013; Debreczeni et al., 2014), which can be used for epidemiological studies and disease control strategies based on restricting the dispersion of viruses by vectors; iii) evaluation of the resistance level to virus accumulation in plant breeding programs (Gil-Salas et al., 2009; Galipienso et al., 2013; Soler et al., 2015); and iv) estimation of fitness in competition and evolutionary experiments (Carrasco et al., 2007; Peña et al., 2014) which can be used for evolutionary and epidemiological studies, as well as for evaluation of resistance durability. HTS can be used for a relative quantification based on the number of reads for the same sequence, but it is still too expensive for these applications.

### Feasibility and Designability

Other important features to be considered in the detection techniques are the costs, throughput screening (number of samples analyzed simultaneously) and easiness, not only during the application but also during the design or development (**Table 3**).

**Table 3:**
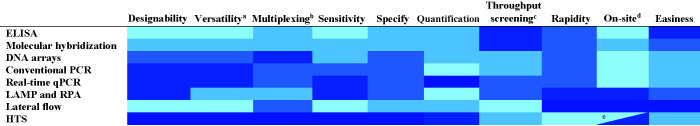
Features of technique types for plant virus detection and diagnostics.

Color intensity is proportional to positive qualification.

ELISA, enzyme-linked immunosorbent assay, PCR, polymerase chain reaction; qPCR, quantitative PCR, LAMP, loop-mediated isothermal amplification, RPA, recombinase polymerase amplification; and HTS, high-throughput sequencing.

a Versatility. Ability for different detection levels (family, genus, species, strain or isolate).

b Multiplexing. Ability to perform parallel analysis (analyze several viruses simultaneously).

c Ability to analyze many samples simultaneously.

d On-site. Ability to detect viruses on field with portable devices.

e Only nanopore sequencing is portable among the HTS techniques.

Sample processing is a critical step and affects the rapidity, easiness and throughput of the detection process. Molecular hybridization and PCR techniques require purification of total RNA or DNA from plants to remove substances inhibiting the detection process (yielding false negatives) or producing a background signal (yielding false positives). Inhibition of PCR can be avoided or minimized by diluting the extracts or by immunocapture (Olmos et al., 2007). HTS also requires purification of DNA or RNA from plants. Libraries can be enriched in viral sequences by using as starting material preparations from which host nucleic acids have been removed by subtractive hybridization or using purified viral particles, double-stranded RNAs (dsRNAs) preparations, which are enriched in replicative intermediates of RNA viruses, or small RNAs resulting from RNA silencing that is a plant response to virus infection (Kreuze et al., 2009; Singh et al., 2012; Kesanakurti et al., 2016; Pecman et al., 2017; Czotter et al., 2018).

Extracts obtained by just grinding plant tissue in buffer can be used for ELISA and LAMP. For some viruses, tissue-prints made by cutting leaf petioles or rolled leaf blades transversely and gently pressing the fresh cut onto nitrocellulose membranes have been analyzed directly with ELISA or molecular hybridization (Narváez et al., 2000; Rubio et al., 2003b; Ferrer et al., 2008). An alternative to passive incubation of probes with the targets immobilized onto membranes in solution is the flow-through hybridization, based on directing a probe flow towards the targets immobilized on the membrane, reducing the hybridization time from hours to a few minutes (Ferriol et al., 2015).

On-site detection of plant viruses is an interesting feature allowing a prompt response. Presently, several techniques are commercially available (Donoso and Valenzuela, 2018). Lateral flow assay (LFA) consists of a chromatographic test strip where crude plant extracts are dropped and move capillarily. The virus is detected when a stained band appears by binding virions with labeled antibodies or viral nucleic acids with labeled DNA or RNA probes (Drygin et al., 2012; Zhang et al., 2014; Koczula and Gallotta, 2016). This procedure takes only about 15 to 30 min. LFA has been used for multiplex detection of potato viruses (Safenkova et al., 2016) and relative quantification (Rohrman et al., 2012). RPA and LAMP isothermal amplification can be performed with crude plant extracts in portable hot blocks and results can be displayed with a portable fluorescence reader or a lateral flow strip so the whole process can take about 45 min. (Zhang et al., 2014; Wilisiani et al., 2019). Oxford Nanopore Technologies has developed MinION, a portable nanopore sequencing device, that can be used for the detection of plant viruses in the field (Boykin et al., 2018; Filloux et al., 2018; Shaffer, 2019), but it is still too expensive for most routine uses.

The ability to develop rapidly new assays is very important given the continuous emergence of new plant viruses. The production of antisera for the serological techniques is lengthy, unpredictable and costly (Boonham et al., 2014). In contrast, the setup of molecular detection methods is a directed process that is cheap, fast and versatile, enabling to address different detection levels and consider the genetic variability of virus populations. Primers and oligoprobes are synthesized and commercialized at a low cost. They can be easily designed with many available software algorithms, such as Prime3 (http://bioinfo.ut.ee/primer3-0.4.0/) and Primer Express (Thermofisher) for PCR, or Primer Explorer (https://primerexplorer.jp/e/), LAMP Designer (Optigene) and LAVA (Torres et al., 2011) for LAMP.

## Disease Management

Eradication or control of virus diseases is difficult given the complex and dynamic nature of virus epidemics and the great evolvability of viruses (Acosta-Leal et al., 2011; Elena et al., 2014). For efficient and durable control, it is necessary to consider the genetic diversity and evolution of virus populations and have specific, fast and reliable diagnostic tools. Disease management in agriculture is based on two approaches: immunization to get resistant plants to viral infections and prophylactic measures to restrain virus dispersion.

### Immunization Measures

Introgression of resistance genes from cultivated or wild species into susceptible related crops by backcrossing (plant breeding) is the most widely-used immunization method. There are two types: i) active resistance driven by resistance proteins, encoded by dominant alleles, which recognize specifically a sequence or conformational pattern of a virus gene (avirulence determinant, Avr) and induces death of the infected cells (hypersensitive response), precluding virus movement to adjacent cells and systemic infection (De Ronde et al., 2014; Peiró et al., 2014); and ii) passive resistance, conferred by resistance recessive alleles encoding host factors critical for viral infection, mostly eukaryotic translation initiation factors (eIF) 4E and 4G, and their isoforms (Truniger and Aranda, 2009; Hashimoto et al., 2016).

Plant breeders usually aim at complete resistance, in which the virus cannot establish a systemic infection. ELISA and molecular hybridization have been used to test germplasm and cultivars for resistance since a good number of plants can be analyzed simultaneously (Rubio et al., 2003a; Soler et al., 2015). When a complete resistance is not possible, breeding for relative or partial resistance (reduction of virus accumulation) or tolerance (reduction of virus damage without affecting virus multiplication) can be a good alternative. The most precise technique to evaluate the level of relative resistance is real-time qPCR (Gil-Salas et al., 2009; Galipienso et al., 2013; Soler et al., 2015). Time-course assays can be used to evaluate relative resistance (that can be measured by ELISA or molecular hybridization) and relative tolerance (measured by observation of symptoms as a proxy of damage). The levels of resistance or tolerance can be estimated as the probability of no infection or no symptoms, respectively, by survival analyses (Kaplan and Meier, 1958).

However, breeding resistant cultivars is unsuitable for many crops and viruses because of the scarcity of resistance genes found in genetically compatible relatives. An alternative may be to change the specificity or range of known resistance genes by artificial evolution so they can confer resistance to novel viruses or virus strains. This approach is based on generating large populations of random mutants from a resistance gene by PCR with a high error rate, followed by the selection of those variants showing the desired resistance properties. Resistance is evaluated by *Agrobacterium*-mediated transient co-expression of each resistance gene mutant and the Avr from the challenge virus in *Nicotiana benthamiana* leaves so the resistance response is observed as a necrotic lesion. This approach has been used to broaden the resistance specificity of the potato gene *Rx* (Farnham and Baulcombe, 2006; Harris et al., 2013), but its use has not become widespread since most mutants are nonfunctional and screening is time-consuming.

Genome editing, like the CRISPR-Cas9 system, could be used to implement in crops the resistance genes obtained by artificial evolution and to mutagenize directly host genes involved in recessive resistance to prevent interaction with viruses (Piron et al., 2010; Chandrasekaran et al., 2016; Pyott et al., 2016). However, the mutagenized plant genes could be involved in important biological functions, so the mutations may also have unexpected adverse effects in plant development or physiology.

Resistance can be ineffective for some virus variants or be overcome by i) interaction with other viruses in mixed infections (Desbiez et al., 2003; García-Cano et al., 2006), ii) positive selection of punctual mutations (Weber et al., 1993; Hébrard et al., 2006; López et al., 2011) or iii) recombination or reassortment events (Qiu and Moyer, 1999; Miras et al., 2014). Plant genes conferring dominant resistance usually target viral protein domains whose function is essential for the virus biology (replication, movement, transmission) and are under strong negative selection. Thus, it is difficult for the virus to fix the mutations producing resistance breakdown. In some cases, overcoming the resistance implemented in a cultivar by plant breeding involves a tradeoff leading to a loss of the virus fitness in non-resistant hosts, thus limiting the cases of emergence and spread of resistance-breaking isolates in the field (García-Arenal and McDonald, 2003). Polygenic resistance is more durable, but resistance implemented in most breeding programs is monogenic because its introgression in the crops is easier (Palloix et al., 2009). Understanding the molecular, evolutionary and epidemiological factors involved in the emergence of resistance-breaking virus isolates is progressing (García-Arenal and McDonald, 2003; Elena et al., 2014), but predicting the durability of new resistances remains elusive and it needs to be tested in the field.

Plant breeders are keen on the host genetic variability, but often they neglect the virus genetic variability, which should be considered when new resistance genes are tested to minimize the possibility of resistance breakdown. Multiplex real-time qPCR is well-suited to evaluate the effect of mixed infections in overcoming resistance. Detection of virus variants with punctual mutations leading to resistance breakdown would be a valuable tool to monitor the dispersion of these variants. However, this has proved to be a difficult task as in most cases resistance breakdown occurs by only one nucleotide substitution (producing one amino acid change). The presence of other neutral substitutions around the critical mutation hinders the design of molecular markers for resistance breakdown. Real-time qPCR with Taqman probes has been developed to detect single nucleotide polymorphisms associated with resistance breakdown for beet necrotic yellow vein virus (BNYVV) and tomato spotted wilt virus (TSWV) (Acosta-Leal and Rush, 2007; di Rienzo et al., 2018);. However, there is no guarantee that these techniques are universal for all isolates of each virus species, since other polymorphic sites nearby can affect the detection process or have epistatic interactions affecting resistance.

Another strategy to obtain resistant plants is based on the RNA silencing mechanism. RNA silencing is a regulatory mechanism of gene expression in eukaryotes and a natural antiviral defense mechanism. The host RNA silencing machinery targets dsRNA that arise from replicative viral intermediates or secondary structures in the genomic RNA due to internal complementarity, which are detected by RNases (Dicer-like proteins) and cleaved into small RNA duplexes (siRNA or miRNAs) of 21–24 nucleotides (nt) in length. One of the two strands of the small RNAs is recruited to the RNA-induced silencing complex (RISC) that subsequently cleaves cognate viral RNAs in a sequence-specific manner. These cleaved RNAs are recognized by RNA dependent RNA polymerase (RDR), which amplifies the dsRNA molecules, thus contributing to the amplification of the host defense mechanism that results in effective inhibition of local and systemic viral infection (Kaldis et al., 2017). To counteract this defensive mechanism, many viruses encode RNA silencing suppressors (Voinnet et al., 1999), which can act in different steps of the silencing pathway, either by binding siRNA duplex or by directly interacting with key components of the RNA silencing machinery. Some synergetic interactions between coinfecting viruses (increasing viral accumulation or symptoms) are caused by the cumulative effect of the silencing suppressors of both viruses (Syller, 2012). Resistance can be obtained by plant transformation, introducing into plants DNA constructs to produce viral dsRNAs or ssRNA with some degree of secondary structure to trigger RNA silencing. Since RNA silencing requires a certain nucleotide identity between the targeted virus and the transgene, it is necessary to evaluate the nucleotide variation of the virus population as explained above. The best technique to evaluate the efficiency of the RNA silencing-based resistances is real-time qPCR.

Silencing resistance breakdown can occur by mutation and selection (de Haan et al., 1992) or by mixed infection with other viruses (Syller, 2012). Transgenic plants with multiple constructs from different viral genomes (from the same species and/or different species) can be used to minimize the risk of resistance breakdown (Bucher et al., 2006; Duan et al., 2012). Another strategy is using a transgene mimicking the secondary structure of endogenous miRNA precursors (involved in plant gene expression and development) to express artificial miRNAS (amiRNAs) targeting viral sequences (Niu et al., 2006; Qu et al., 2007; Liu et al., 2017). The main advantage is that the short sequence of amiRNAs makes it easier to find conserved sequences that are more difficult to overcome (they are usually under strong negative selective pressure) and can be used for broad range targets (genera and families). However, the amiRNA resistance can be also overcome by mutation and selection (Simón-Mateo and García, 2006; Lin et al., 2009; Lafforgue et al., 2011) or interaction with co-infecting viruses (Pacheco et al., 2012; Martínez et al., 2013). A strategy to obtain more durable resistances is to express multiple amiRNAs to target different regions within a single viral genome (Fahim et al., 2012; Kung et al., 2012; Lafforgue et al., 2013; Kis et al., 2016). Synthetic trans-acting small interfering RNAs (syn-tasiRNAs) is another class of artificial small RNAs engineered in plants, which are especially suited to target multiple sites within a viral genome or different unrelated viruses (Carbonell et al., 2016; Chen et al., 2016; Carbonell and Daròs, 2019; Carbonell et al., 2019). The durability of these resistances can be evaluated by experimental evolution based on successive passages of the virus in the resistant plants. Resistance-breakdown can be detected by qPCR as an increase in virus accumulation (Carrasco et al., 2007; Peña et al., 2014). The mutations fixed after the passages can be detected by PCR followed by cloning and Sanger sequencing or HTS. Identification of the mutations leading to resistance breakdown is possible if infectious clones exist, so that each mutation can be tested for the increase of virus accumulation. An alternative to transgenic plants is the exogeneous application of *in vitro*-produced dsRNAs from viral sequences onto plants (Tenllado and Díaz-Ruiz, 2001; Kaldis et al., 2017; Niehl et al., 2018). The efficacy of this technique of immunization can be improved by high-pressure spraying plants (Dalakouras et al., 2016), using cell-penetrating peptides (Numata et al., 2014) or clay nanoparticles stabilizing dsRNAs (Mitter et al., 2017).

Another immunization method is cross-protection based on inoculating mild or attenuated viral strains to protect plants against severe strains of the same virus. Cross-protection has been applied to several viruses and crops, such as PepMV in tomato and CTV in citrus crops (Pechinger et al., 2019). The mechanism of cross-protection is poorly understood and several models have been proposed: prevention of virus entry into cells; competition for host factors for replication, interference with disassembly, translation or replication; and induction of RNA silencing leading to sequence-specific degradation of the superinfecting virus (Ziebell and Carr, 2010; Folimonova, 2013). It has been suggested that cross-protection in some viruses might be an active virus-controlled function involving virus-coded proteins (Folimonova, 2013). A recent model proposed that cross-protection is a mechanism that prevents the virus progeny to replicate in the cells to minimize mutation rate and collaterally targets highly homologous superinfecting viruses that are indistinguishable from progeny viruses (Zhang X. et al., 2018). To apply cross-protection it is necessary to evaluate the genetic and biological variability of the local virus population and search for mild isolates genetically close to the severe ones (Hanssen et al., 2010). Mild or attenuated strains can also be generated by thermal treatment, random mutagenesis by using chemicals as nitrous acids and selection or directed mutagenesis in viral RNA silencing suppressors (Ziebell and Carr, 2010). Cross-protection assays can be evaluated by real-time qPCR with probes specific for each virus variant (Ruiz-Ruiz et al., 2009b; Hanssen et al., 2010) and SSCP analysis (Sambade et al., 2002). However, the protection exerted by the mild isolate can be overcome and even a more severe disease can emerge by transmission to a different host species, interaction with other viruses in mixed infections, or recombination between divergent strains or viruses (Fulton, 1986). Therefore cross-protection should be used only for devastating diseases when other protection measures fail, and the process should be monitored closely.

### Prophylactic Measures

Quarantine (control of borders) and sanitary certification of virus-free germplasm (seeds or asexual propagative tissues) are the first measures to prevent the introduction and emergence of new viruses in a geographical area. Virus detection should be based on sensitive and broad-spectrum methods, since discarding healthy material is preferable to the risk of spreading new diseases. On-site detection devices can be useful to make decisions quickly, thus preventing importation and exportation delays. HTS is the most powerful detection procedure since it can detect all the viruses (known and unknown) present in a plant in an unbiased way. Its use in quarantine and clean plant programs is increasing as it is becoming more economically affordable (Villamor et al., 2019). Phytosanitary certificates should be based on propagative material free from only harmful viruses, since plants can harbor many viruses (Maree et al., 2018).

**Figure 4:**
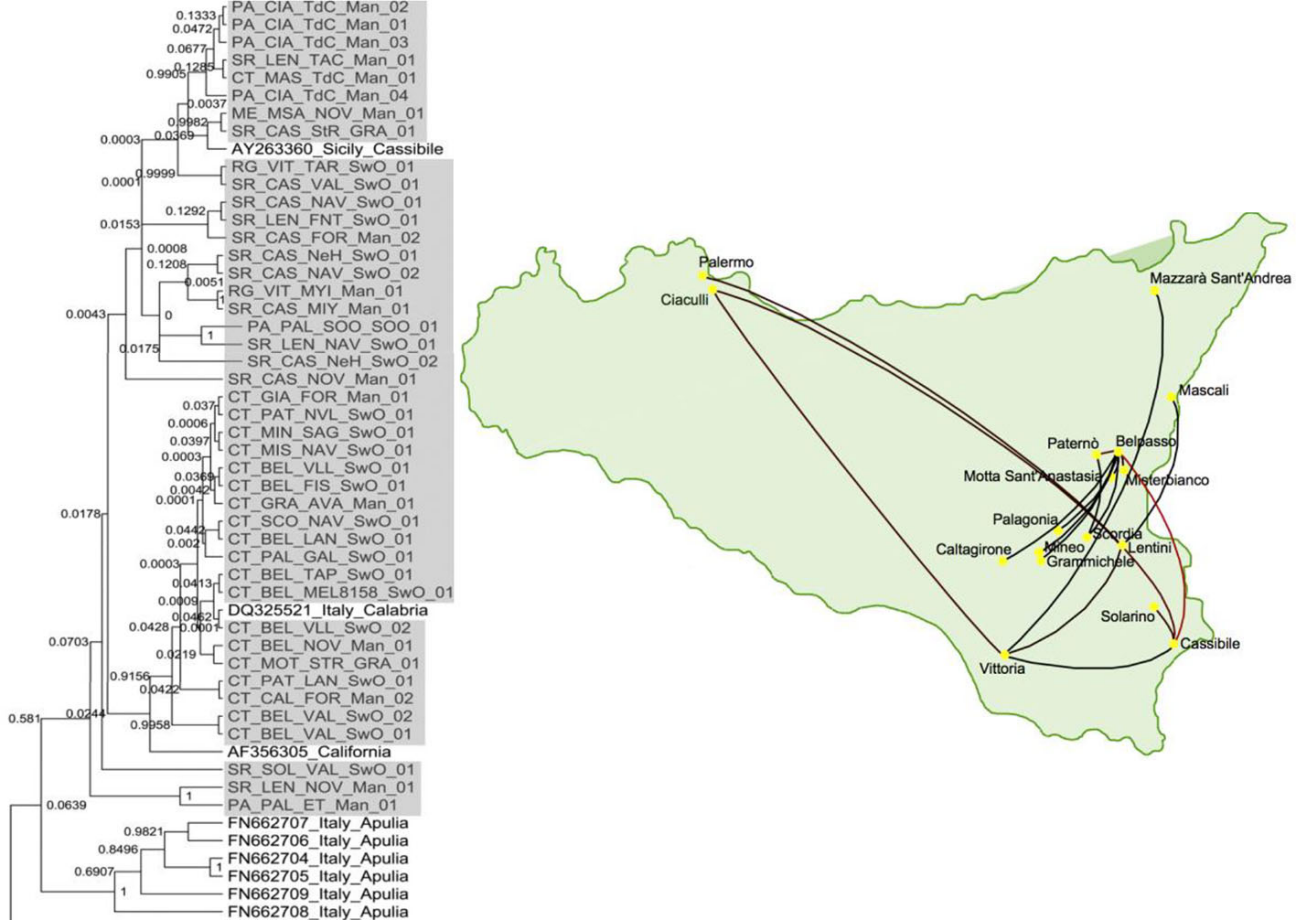
Phylogenetic tree of citrus tristeza virus (CTV) isolates collected from the first outbreak of CTV in Sicily, Italy and a map showing the migration paths within Sicily (Davino et al., 2013).

Since epidemiology and evolution are coupled in viruses, phylogeographic studies, comparing genetic variation in space and time, can provide useful information on the introduction sites of new viruses and the dispersal paths (Gómez et al., 2012; Davino et al., 2013). As an illustration, **Figure 4** shows the migration paths of one of the strains in the first introduction of CTV in Sicily, Italy (Davino et al., 2013), which were inferred by Bayesian phylogeographic analysis with the program BEAST v1.6.2 (Drummond and Rambaut, 2007). Phylogenetic analyses showed that these Sicilian CTV isolates were genetically close to CTV isolates from mainland Italy and California.

Sanitation, that is, removing viruses from valuable cultivars, is necessary when no healthy plants are available. Virus-free plants are usually produced by thermotherapy, chemotherapy, electrotherapy, and tissue culture alone or combined (Naik and Buckseth, 2018). Thermotherapy could inactivate viruses by viral RNA breakage, viral particle disruption or coat protein rupture, viral replicase inactivation, virus movement inhibition and/or translation reduction (Hull, 2002). Chemotherapy is based on antiviral drugs to inhibit or disrupt specific steps of the virus life cycle, e.g., nucleoside analogs inhibiting replication and protease inhibitors preventing protein processing. Antiviral drugs are costly and are used only to regenerate healthy mother plants for vegetative propagation or seed production (Panattoni et al., 2013). The sanitated plants must be evaluated and confirmed to be virus-free with very sensitive techniques such as real-time qPCR. Nucleoside analogs can also be used to increase the already high mutation rate of RNA viruses, so that the excess of mutations would lead to a loss of genetic information and virus extinction (lethal mutagenesis or error catastrophe). This approach has been assayed recently with a plant virus, tobacco mosaic virus (TMV), resulting in a loss of viral infectivity (Díaz-Martínez et al., 2018).

An agronomical practice to limit virus dispersal consists of removing virus-infected plants from crops or weeds acting as inoculum source. This requires rapid and specific detection techniques able to analyze many samples from the field, such as ELISA and molecular hybridization, especially using tissue-prints (Rubio et al., 1999; Rubio et al., 2003b). Roguing is effective only if the virus incidence is low after a recent introduction or in isolated areas. Other practices consist of interrupting the virus transmission chain. Many seed-borne viruses (carried on the seed coat) can be removed with chemical disinfectants such as sodium hypochlorite, trisodium phosphate, hydrochloric acid and ozone, whereas some seed-transmitted viruses (infecting the seed embryo) can be eliminated by thermotherapy (Ling, 2010; Paylan et al., 2014). Multiplex (RT)-PCR to detect simultaneously the seed-transmitted and seed-borne viruses for a crop can be a useful tool (Panno et al., 2012). Incidence of plant viruses mechanically transmissible by contact, like those of the genus *Tobamovirus*, can be reduced by hygienic measures such as using disposable gloves or washing hands with disinfectant, heat sterilization of tools and debris and limiting the access to crops (Broadbent, 1976). Most plant viruses are transmitted by arthropod vectors, mainly aphids, whiteflies, and thrips. Three strategies to prevent the spread of plant viruses by vectors have been used (Antignus, 2012; Fereres and Raccah, 2015): i) Reducing vector populations by pesticides; biological control with natural enemies such as arthropod predators and parasitoids (Téllez et al., 2017) or entomopathogenic fungi, nematodes, bacteria and viruses (Kalha et al., 2014); and biotechnology-based approaches based on protease inhibitors, neurotoxins, or RNA silencing of genes essential for insect development or metabolism (Fereres and Raccah, 2015; Nandety et al., 2015; Vogel et al., 2019). ii) Preventing the vector from reaching the crop with barriers (greenhouses and barrier plants), by interference of the insect vision with UV-absorbing plastics and reflective surfaces, by agronomical practices such as changing the planting or sowing dates to avoid high vector populations, or imposing a time gap between crops and/or space gap between plots to break the transmission cycle (Antignus, 2012; Fereres and Raccah, 2015). iii) Interfering with the transmission process by spraying mineral oils, synthetic peptides or modified proteins to outcompete virus-encoded proteins needed for virus attachment to insect receptors (Lecoq and Desbiez, 2012; Blanc et al., 2014).

The rate of insect transmission can be evaluated using different detection techniques, such as ELISA, molecular hybridization and PCR, to detect the virus in the receptor plants and real-time qPCR to estimate the virus titer in the source plants, which affects transmissibility (Olmos et al., 2005; Rotenberg et al., 2009; Ferriol et al., 2013; Debreczeni et al., 2014).

## Conclusions and Future Trends

The main challenge of agriculture in this century is to produce nutritious food for the growing world population in a sustainable manner while protecting the environment and human health (Pretty, 2008; Pretty et al., 2010). Damages caused by pests and diseases have a considerable negative economic impact in agriculture, being emergent viral diseases particularly important (Anderson et al., 2004; Mumford et al., 2016).

**Figure 5:**
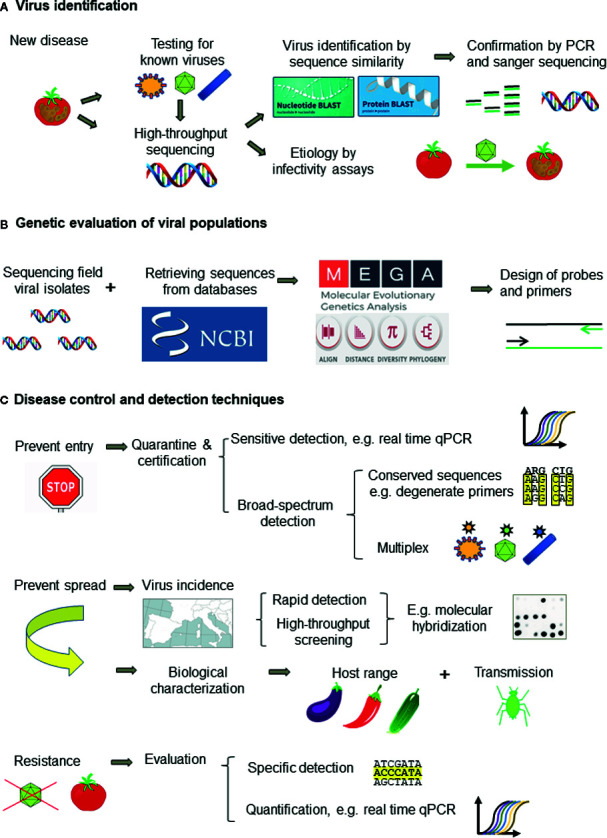
Workflow for tackling plant viral diseases showing: **(A)** the approach for plant virus identification, **(B)** genetic evaluation of virus populations for designing detection techniques, and **(C)** the appropriate detection techniques for disease management.

The correct identification of viruses is critical for disease management. However, the great ability of viruses to evolve and generate molecular and biological variation is a major difficulty for virus detection and disease management. Presently, when a new virus-like disease appears, the first approach is to test for known viruses with well-established techniques (**Figure 5A**). ELISA is the most popular for routine analysis because of historical reasons, easiness to perform with little training and commercial availability of antibodies specific for the main plant viruses. However, antibody production is expensive, time-consuming, and unpredictable, and it cannot be designed to cope with viral variability. In contrast, the development of molecular techniques is fast and cheap, making them more appropriate to cope with the frequent cases of new virus emergence (Boonham et al., 2014). PCR techniques are the most widely used because of the easy design, versatility, and low cost of primers. Real-time qPCR is becoming the molecular method of choice for routine virus analysis (especially for new viruses for which antibodies are not available or with low accumulation levels) and quantification. On-site detection techniques by LFA and isothermal amplification (RPA and LAMP) allow an almost immediate response and are rapidly developing. When these techniques fail to detect the virus causing disease, the best approach is to use HTS, which can identify all the viruses in one plant, albeit infectivity assays or field surveys are necessary to determine which of the viruses detected is likely the disease causal agent (**Figure 5A**). In some cases, such as quarantine, using HTS as first approach for virus detection can be more profitable than testing many viruses with ELISA or molecular detection techniques. However, HTS is still too expensive for most routine analyses and it is necessary to develop rapid and accurate detection techniques for each virus, being PCR the easiest to develop. The design of primers or probes for accurate detection requires to evaluate the genetic diversity of viral populations by analysis of nucleotide sequences. (**Figure 5B**).

Presently, disease management relies on preventing introduction of new viruses by border control and certification of virus-free propagative material (e.g., seeds) and preventing virus dispersal in the field. Quarantine and certification require sensitive and broad-spectrum detection methods to minimize escapes such as the use of conserved primers specific for virus genera or families and multiplex procedures (**Figure 5C**). HTS is the best procedure and it is becoming affordable in quarantine facilities given the devastating consequences of introducing new pathogens. Prophylactic measures to prevent or minimize virus dispersal require information on the virus biology (host range, transmission way, etc.), virus incidence and epidemiology. Obtaining this information needs using high-throughput screening techniques able to analyze a high number of samples such as ELISA and molecular hybridization (**Figure 5C**). The other keystone for plant disease control is the obtention of resistant cultivars, which is performed mainly by plant breeding and commercialized by seed companies. However, apart from the important effort involved in breeding programs, resistance is not available for most crops and viruses. Genetic engineering, despite the great potential and some remarkable successes (Fuchs and Gonsalves, 2007), faces heavy opposition in some countries due to the public concern on the potential ecological impact of transgenic plants (Jones, 2006). Resistant plants should be evaluated with specific methods, as resistance depends on host and virus genotypes. Partial resistance can be evaluated by real-time qPCR (**Figure 5C**). Genome editing by CRISPR and the application of dsRNA-loaded clay particles to trigger RNA silencing are promising research fields. In any case, to aim at more durable and effective protection, it is necessary to characterize the genetic variability and relationships of plant viruses, as well as the factors and mechanisms involved in genetic change.

HTS excels for its broad-spectrum and multiplex detection, sensitivity, and precise quantification. No previous knowledge is required, enabling unbiased detection and discovery of new viruses. The sequences obtained allow a precise taxonomic assignation and an estimation of genetic relationships with other viruses and viral isolates. It is reasonable to expect that HTS, especially portable nanopore sequencing devices, will become the standard diagnostic procedure as costs will be dropping and analytical procedures improving.

## Author Contributions

LR conceived the idea. LR, IF, and LG wrote the manuscript. LR and IF designed the figures. All authors contributed to the article and approved the submitted version.

## Funding

This work was partially funded by grants RTA2017-00061-C03-02 from Spanish Ministerio de Ciencia e Innovación co-financed by FEDER and 51912 from IVIA. IF acknowledges financial support from the Spanish Ministerio de Economia y Competitividad, through the “Severo Ochoa Programme for Centers of Excellence in R&D” 2016-2019 (SEV-2015-0533).

## Disclaimer

The opinions expressed, and arguments employed in this paper are the sole responsibility of the authors and do not necessarily reflect those of the OECD or of the governments of its Member countries.

## Conflict of Interest

The authors declare that the research was conducted in the absence of any commercial or financial relationships that could be construed as a potential conflict of interest.
