# Supplementary material for: Detection of Plant Viruses and Disease Management: Relevance of Genetic Diversity and Evolution
Source: Front Plant Sci. 2020 Jul 17;11:1092. doi: 10.3389/fpls.2020.01092 (PMC7380168; doi:10.3389/fpls.2020.01092)
Supplement: Supplementary file 1 [file Presentation_1.pdf]

SUPPLEMENTARY DATA

TABLE S1. Hybridization with single probes specific to plant virus species

**Family**	**Genus**	**Virus**	**References**
Alphaflexiviridae	*Potexvirus*	*Cymbidium mosaic virus* (CyMV)	Chia et al., 1992
Alphaflexiviridae	*Potexvirus*	*Papaya mosaic virus* (PMV)	Roy et al., 1988
Alphaflexiviridae	*Potexvirus*	*Potato virus X* (PVX)	Baulcombe et al., 1984; Nikolaeva et al., 1990
Amalgamaviridae	*Amalgavirus*	*Southern tomato virus* (STV)	Puchades et al., 2017
Betaflexiviridae	*Carlavirus*	*Potato virus M* (PVM)	Nikolaeva et al., 1990
Betaflexiviridae	*Carlavirus*	*Potato virus S* (PVS)	Nikolaeva et al., 1990
Betaflexiviridae	*Citrivirus*	*Citrus leaf blotch virus* (CLBV)	Galipienso et al., 2004
Bromoviridae	*Cucumovirus*	*Cucumber mosaic virus* (CMV)	Juan et al., 2007
Bromoviridae	*Ilarvirus*	*Apple mosaic virus* (ApMV)	Saade et al., 2000
Bromoviridae	*Ilarvirus*	*Parietaria moltle virus* (PMoV)	Galipienso et al., 2005
Bromoviridae	*Ilarvirus*	*Prune dwarf virus* (PDV)	Saade et al., 2000
Bromoviridae	*Ilarvirus*	*Prunus necrotic ringspot virus* (PNRSV)	Sánchez et al., 1998
Bromoviridae	*Ilarvirus*	*Tobacco streak virus* (TSV)	Stenger et al., 1987
Caulomoviridae	*Badnavirus*	*Citrus yellow mosaic virus* (CYMV)	Gopi et al., 2010
Closteroviridae	*Ampelovirus*	*Grapevine leafroll-associated closterovirus 3* (GLRaV-3)	Saldarelli et al., 1994
Closteroviridae	*Closterovirus*	*Citrus tristeza virus* (CTV)	Narváez et al., 2000
Geminiviridae	*Begomovirus*	*Abutilon mosaic virus* (AbMV)	Horns and Jeske, 1991
Geminiviridae	*Begomovirus*	*Tomato leaf curl New Delhi virus* (ToLCNDV)	Alfaro-Fernández et al., 2016
Geminiviridae	*Begomovirus*	*Tomato yellow leaf curl Sardinia virus* (TYLCSV)	Alfaro-Fernández et al., 2016
Geminiviridae	*Begomovirus*	*Tomato yellow leaf curl virus* (TYLCV)	Alfaro-Fernández et al., 2016
Luteoviridae	*Luteovirus*	*Barley yellow dwarf virus* (BYDV)	Figueira et al., 1997
Ophioviridae	*Ophiovirus*	*Citrus psorosis virus* (CPsV)	Martín et al., 2004
Potyviridae	*Ipomovirus*	*Cucumber vein yellowing virus* (CVYV)	Rubio et al., 2003
Potyviridae	*Potyvirus*	*Bean common mosaic virus* (BCMV)	Bijaisoradat and Kuhn, 1988
Potyviridae	*Potyvirus*	*Bean yellow mosaic virus* (BYMV)	Tracy et al., 1991
Potyviridae	*Potyvirus*	*Clover yellow vein virus* (CYVV)	Tracy et al., 1991
Potyviridae	*Potyvirus*	*Papaya ringspot virus* (PRSV)	Juan et al., 2007
Potyviridae	*Potyvirus*	*Peanut mottle virus* (PeMoV)	Bijaisoradat and Kuhn, 1988
Potyviridae	*Potyvirus*	*Plum pox virus* (PPV)	Palkovics et al, 1994
Potyviridae	*Potyvirus*	*Potato virus Y* (PVY)	Nikolaeva et al., 1990
Potyviridae	*Potyvirus*	*Watermelon mosaic virus* (WMV)	Juan et al., 2007
Potyviridae	*Potyvirus*	*Zuccini yellow mosaic virus* (ZYMV)	Juan et al., 2007
Secoviridae	*Comovirus*	*Squash mosaic virus* (SqMV)	Juan et al., 2007
Secoviridae	*Fabavirus*	*Broad bean wilt virus 1* (BBWV-1)	Ferriol et al., 2015
Secoviridae	*Fabavirus*	*Broad bean wilt virus 2* (BBWV-2)	Ferriol et al., 2015
Secoviridae	*Fabavirus*	*Cucurbit mild mosaic virus* (CuMMV)	Ferriol et al., 2015
Secoviridae	*Fabavirus*	*Gentian mosaic virus* (GeMV)	Ferriol et al., 2015
Secoviridae	*Fabavirus*	*Lamium mild mosaic virus* (LMMV)	Ferriol et al., 2015
Secoviridae	*Nepovirus*	*Arabis mosaic virus* (ArMV)	Jelkman et al., 1988
Tombusviridae	*Carmovirus*	*Carnation mottle virus* (CarMV)	Sánchez-Navarro et al., 1996
Tombusviridae	*Carmovirus*	*Melon necrotic spot virus* (MNSV)	Gosalvez et al., 2003
Tospoviridae	*Orthotospovirus*	*Tomato spotted wilt virus* (TSWV)	Huguenot et al., 1990
Virgaviridae	*Tobamovirus*	*Cucumber green mottle mosaic virus* (CGMMV)	Francki and Palukaitis, 1986
Virgaviridae	*Tobamovirus*	*Odontoglossum ringspot virus* (ORSV)	Chia et al., 1992
Virgaviridae	*Tobamovirus*	*Tobacco mosaic virus* (TMV)	Sela et al., 1984
Virgaviridae	*Tobravirus*	*Tobacco rattle virus* (TRV)	Van der Vlugt et al., 1988

TABLE S2. PCR assays with conserved primers for plant virus families and genera

Family	Genera
Alphaflexiviridae	*Potexvirus* (van der Vlugt et al., 2002)
Aspiviridae	*Ophiovirus* (Vaira et al., 2003)
Bromoviridae (Untiveros et al., 2010)	*Bromovirus* + *Cucumovirus* (Seo et al., 2014)<br>*Cucumovirus* (Choi et al., 1999)<br>*Ilarvirus* (Untiveros et al., 2010; Candresse et al., 1997; Saade et al., 2000)
Betaflexiviridae	*Carlavirus* (Badge et al., 1996; Nie et al., 2008)<br>*Capillovirus* (Foissac et al., 2005)<br>*Foveavirus* (Dovas and Katis, 2003b; Foissac et al., 2005)<br>*Trichovirus* (Saldarelli et al., 1998; Foissac et al., 2005)<br>*Vitivirus* (Dovas and Katis, 2003b)
Caulimoviridae	*Badnavirus* (Thomson et al., 1996),<br>*Caulimovirus* (Pappu and Druffel, 2009)
Closteroviridae	*Ampelovirus* (Maliogka et al., 2008),<br>*Crinivirus* + *Closterovirus* (Tian et al., 1996)<br>*Closterovirus* (Karasev et al., 1994; Saldarelli et al., 1998; Dovas and Katis, 2003a),<br>*Crinivirus* (Wintermantel and Hladky, 2010)
Fimoviridae (Elbeaino et al., 2013)	
Geminiviridae	*Begomovirus* (Rojas, 1993; Deng et al., 1994; Reddy et al., 2005),<br>*Mastrevirus* (Rybicki and Hughes, 1990; Willment et al., 2001)
Luteoviridae (Chomič et al., 2010)	*Polerovirus* (Lotos et al., 2014)<br>*Luteovirus* + *Polerovirus* (Robertson et al., 1991)
Potyviridae (Gibbs and Mackenzie, 1997; Chen and Adams, 2001)	*Potyvirus* (Pappu et al., 1993; Colinet et al., 1994; Van der Vlugt et al., 1999; Hsu et al., 2005; Ha et al., 2008; Zheng et al., 2010)
Secoviridae	*Comovirus* + *Fabavirus* + *Nepovirus* (Maliogka et al., 2004),<br>*Fabavirus* (Panno et al., 2014)<br>*Nepovirus* (Wetzel et al., 2002)<br>*Torradovirus* (Verbeek et al., 2012)
Tombusviridae	*Betacarmovirus* + *Dianthovirus* + *Tombusvirus* (Morozov et al., 1995)
Tospoviridae (Mumford et al., 1996; Chu et al., 2001; Okuda and Hanada, 2001; Hassani-Mehraban et al., 2016; Bald-Blume et al., 2017)	
Tymoviridae (Sabanadzovic et al., 2000)	
Virgaviridae	*Tobamovirus* (Letschert et al., 2002; Dovas et al., 2004; Li et al., 2018)

TABLE S3. LAMP assays for plant viruses

Family	Genus	Virus	Reference
Alphaflexiviridae	*Potexvirus*	*Cymbidium mosaic virus* (CymMV)	Lee et al., 2011
		Pepino mosaic virus (PepMV)	Bhat et al., 2013
Amalgaviridae	*Amalgavirus*	*Southern tomato virus* (STV)	Elvira-Gonzalez et al., 2017
Betaflexiviridae	*Carlavirus*	*Lily symptomless virus* (LSV)	He et al., 2016
Bromoviridae	*Cucumovirus*	*Cucumber mosaic virus* (CMV)	Bhat et al., 2013
		Tomato aspermy virus (TAV)	Liu et al., 2010a
	Ilarvirus	*Prunus necrotic ringspot virus* (PNRSV)	Zong et al., 2014
Caulimoviridae	*Badnavirus*	*Banana streak virus* (BSV)	Peng et al., 2012a
		Piper yellow mottle virus (PYMV)	Bhat et al., 2013
	Tungrovirus	*Rice tungro bacilliform virus* (RTBV)	Le et al., 2010
Closteroviridae	*Closterovirus*	*Citrus tristeza virus* (CTV)	Ghosh et al., 2019
	Crinivirus	*Cucurbit chlorotic yellows virus* (CCYV)	Wang et al., 2014
		Tomato chlorosis virus (ToCV)	Kil et al., 2015
Geminiviridae	*Begomovirus*	*Tomato leaf curl Sinaloa virus* (ToLCSiV	Herrera-Vásquez et al., 2018
		Potato yellow mosaic Panama virus (PYMPV)	Herrera-Vásquez et al., 2018
		Tomato leaf curl Bangalore virus (ToLCBaV)	Arutselvan et al., 2017
		Tomato yellow mottle virus (TYMoV)	Herrera-Vásquez et al., 2018
		Tomato yelow leaf curl virus (TYLCV)	Fukuta et al., 2003b
		Squash leaf curl virus (SLCV)	Kuan et al., 2010
Luteoviridae	*Luteovirus*	*Barley yellow dwarf virus* (BYDV)	Zhao et al., 2010
	Polerovirus	*Potato leafroll virus* (PLRV)	Leone et al., 1997; Ahmadi et al., 2013
Nanoviridae	*Babuvirus*	*Banana bunchy top virus* (BBTV)	Peng et al., 2012b
Phenuiviridae	*Tenuivirus*	*Rice grassy stunt virus* (RGSV)	Le et al., 2010
		Rice stripe virus (RSV)	Le et al., 2010
Potyviridae	*Bymovirus*	*Wheat yellow mosaic virus* (WYMV)	Zhang et al., 2011
	Potyvirus	*Sugarcane mosaic virus* (SCMV)	He et al., 2016
		Japanese yam mosaic virus (JYMV)	Fukuta et al., 2003a
		Plum pox virus (PPV)	Nie, 2005; Varga and James, 2006; Olmos et al., 2007; Zhao et al., 2012
		Potato virus Y (PVY)	Przewodowska et al., 2015
		Sorghum mosaic virus (SrMV)	He et al., 2016
		Tobacco etch virus (TEV)	Zhao et al., 2012
		Tobacco vein banding mosaic virus (TVBMV)	Zhao et al., 2012
Reoviridae	*Fijivirus*	*Rice black-streaked dwarf virus* (RBSDV)	Le et al., 2010
		Southern rice black-streaked dwarf virus (SRBSDV)	Zhou et al., 2012
	Oryzavirus	*Rice ragged stunt virus* (RRSV)	Le et al., 2010
	Phytoreovirus	*Rice gall dwarf virus* (RGDV)	Le et al., 2010
		Rice dwarf virus (RDV)	Le et al., 2010
Rhabdoviridae	*Nucleorhabdovirus*	*Rice transitory yellowing virus* (RTYV)	Le et al., 2010
Secoviridae	*Comovirus*	*Bean pod mottle virus* (BPMV)	Wei et al., 2012
	Torradovirus	*Tomato torrado virus* (ToTV)	Budziszewska et al., 2016
Sequiviridae	*Waikavirus*	*Rice tungro spherical virus* (RTSV)	Le et al., 2010
Tospoviridae	*Orthotospovirus*	*Tomato spotted wilt virus* (TSWV)	Fukuta et al., 2004
Virgaviridae	*Tobamovirus*	*Tobacco mosaic virus* (TMV)	Liu et al., 2010b

REFERENCES

Ahmadi, S., Almasi, M. A., Fatehi, F., Struik, P. C., and Moradi, A. (2013). Visual detection *of Potato leafroll virus* by one-step reverse transcription loop-mediated isothermal amplification of DNA with hydroxynaphthol blue dye. *J. Phytopathol.* 161, 120-124.

Alfaro-Fernández, A., Sánchez-Navarro, J., Landeira, M., Font, M., Hernández-Llópis, D., and Pallás, V. (2016). Evaluation of PCR and non-radioactive molecular hybridization techniques for the routine diagnosis of *Tomato leaf curl New Delhi virus*, *Tomato yellow leaf curl virus* and *Tomato yellow leaf curl Sardinia virus. J. Plant Pathol.* 98, 245-254.

Arutselvan, R., Reddy, M. K., and Makeshkumar, T. (2017). Rapid detection of tomato leaf curl Bengalore virus through loop mediated isothermal amplification assay *VirusDisease* 28, 303-308.

Badge, J., Brunt, A., Carson, R., Dagless, E., Karamagioli, M., Phillips, S., et al. (1996). A carlavirus-specific PCR primer and partial nucleotide sequence provides further evidence for the recognition of cowpea mild mottle virus as a whitefly-transmitted carlavirus. *Eur. J. Plant Pathol.* 102, 305-310.

Bald-Blume, N., Bergervoet, J. H., and Maiss, E. (2017). Development of a molecular assay for the general detection of tospoviruses and the distinction between tospoviral species. *Arch. Virol.* 162, 1519-1528.

Baulcombe, D., Flavell, R., Boulton, R., and Jellis, G. (1984). The sensitivity and specificity of a rapid nucleic acid hybridization method for the detection of potato virus X in crude sap samples. *Plant Pathol.* 33, 361-370.

Bhat, A., Siljo, A., and Deeshma, K. (2013). Rapid detection of *Piper yellow mottle virus* and *Cucumber mosaic virus* infecting black pepper (*Piper nigrum*) by loop-mediated isothermal amplification (LAMP). *J. Virol. Methods* 193, 190-196.

Bijaisoradat, M., and Kuhn, C. (1988). Detection of two viruses in peanut seeds by complementary DNA hybridization tests. *Plant Dis.* 72, 956-959.

Budziszewska, M., Wieczorek, P., and Obrępalska-Stęplowska, A. (2016). One-step reverse transcription loop-mediated isothermal amplification (RT-LAMP) for detection of tomato torrado virus. *Arch. Virol.* 161, 1359-1364.

Candresse, T., Kofalvi, S., Lanneau, M., and Dunez, J. (1997). A PCR-ELISA procedure for the simultaneous detection and identification of prunus necrotic ringspot (PNRSV) and apple mosaic (ApMV) ilarviruses. *Acta Hortic.* 472, 219-226.

Chen, J., and Adams, M. (2001). A universal PCR primer to detect members of the *Potyviridae* and its use to examine the taxonomic status of several members of the family. *Arch. Virol.* 146, 757-766.

Chia, T., Chan, Y., and Chua, N. (1992). Detection and localization of viruses in orchids by tissue-print hybridization. *Plant Pathol.* 41, 355-361.

Choi, S. K., Choi, J. K., Park, W. M., and Ryu, K. H. (1999). RT-PCR detection and identification of three species of cucumoviruses with a genus-specific single pair of primers. *J. Virol. Methods* 83, 67-73.

Chomič, A., Pearson, M., Clover, G., Farreyrol, K., Saul, D., Hampton, J., et al. (2010). A generic RT-PCR assay for the detection of *Luteoviridae. Plant Pathol.* 59, 429-442.

Chu, F., Chao, C., Chung, M., Chen, C., and Yeh, S. (2001). Completion of the genome sequence of *Watermelon silver mottle virus* and utilization of degenerate primers for detecting tospoviruses in five serogroups. *Phytopathology* 91, 361-368.

Colinet, D., Kummert, J., Lepoivre, P., and Semal, J. (1994). Identification of distinct potyviruses in mixedly-infected sweet potato by the polymerase chain reaction with degenerate primers. *Phytopathology* 84, 65-69.

Deng, D., McGrath, P., Robinson, D., and Harrison, B. (1994). Detection and differentiation of whitefly-transmitted geminiviruses in plants and vector insects by the polymerase chain reaction with degenerate primers. *Ann. Appl. Biol.* 125, 327-336.

Dovas, C. I., Efthimiou, K., and Katis, N. I. (2004). Generic detection and differentiation of tobamoviruses by a spot nested RT-PCR-RFLP using dI-containing primers along with homologous dG-containing primers. *J. Virol. Methods* 117, 137-144.

Dovas, C., and Katis, N. (2003a). A spot multiplex nested RT-PCR for the simultaneous and generic detection of viruses involved in the aetiology of grapevine leafroll and rugose wood of grapevine. *J. Virol. Methods* 109, 217-226.

Dovas, C. I., and Katis, N. I. (2003b). A spot nested RT-PCR method for the simultaneous detection of members of the *Vitivirus* and *Foveavirus* genera in grapevine. *J. Virol. Methods* 107, 99-106.

Elbeaino, T., Whitfield, A., Sharma, M., and Digiaro, M. (2013). *Emaravirus*-specific degenerate PCR primers allowed the identification of partial RNA-dependent RNA polymerase sequences of Maize red stripe virus and Pigeonpea sterility mosaic virus. *J. Virol. Methods* 188, 37-40.

Elvira-González, L., Puchades, A., Carpino, C., Alfaro-Fernández, A., Font-San-Ambrosio, M., Rubio, L., et al. (2017). Fast detection of *Southern tomato virus* by one-step transcription loop-mediated isothermal amplification (RT-LAMP). *J. Virol. Methods* 241, 11-14.

Ferriol, I., Rangel, E., Panno, S., Davino, S., Han, C., Olmos, A., et al. (2015). Rapid detection and discrimination of fabaviruses by flow-through hybridisation with genus-and species-specific riboprobes. *Ann. Appl. Biol.* 167, 26-35.

Figueira, A. R., Domier, L. L., and D'Arcy, C. J. (1997). Comparison of techniques for detection of barley yellow dwarf virus-PAV-IL. *Plant Dis.* 81, 1236-1240.

Foissac, X., Svanella-Dumas, L., Gentit, P., Dulucq, M., Marais, A., and Candresse, T. (2005). Polyvalent degenerate oligonucleotides reverse transcription-polymerase chain reaction: a polyvalent detection and characterization tool for trichoviruses, capilloviruses, and foveaviruses. *Phytopathology* 95, 617-625.

Francki, R. I., Hu, J., and Palukaitis, P. (1986). Taxonomy of cucurbit-infecting tobamoviruses as determined by serological and molecular hybridization analyses. *Intervirology* 26, 156-163.

Fukuta, S., Iida, T., Mizukami, Y., Ishida, A., Ueda, J., Kanbe, M., et al. (2003a). Detection of Japanese yam mosaic virus by RT-LAMP. *Arch. Virol.* 148, 1713-1720.

Fukuta, S., Kato, S., Yoshida, K., Mizukami, Y., Ishida, A., Ueda, J., et al. (2003b). Detection of tomato yellow leaf curl virus by loop-mediated isothermal amplification reaction. *J. Virol. Methods* 112, 35-40.

Fukuta, S., Ohishi, K., Yoshida, K., Mizukami, Y., Ishida, A., and Kanbe, M. (2004). Development of immunocapture reverse transcription loop-mediated isothermal amplification for the detection of tomato spotted wilt virus from chrysanthemum. *J. Virol. Methods* 121, 49-55.

Galipienso, L., Carmen Vives, M., Navarro, L., Moreno, P., and Guerri, J. (2004). Detection of *Citrus leaf blotch virus* using digoxigenin-labeled cDNA probes and RT–PCR. *Eur. J. Plant Pathol.* 110, 175-181.

Galipienso, L., Herranz, M., Pallás, V., and Aramburu, J. (2005). Detection of a tomato strain of *Parietaria mottle virus* (PMoV-T) by molecular hybridization and RT-PCR in field samples from north-eastern Spain. *Plant Pathol.* 54, 29-35.

Ghosh, D. K.; Warghane, A.; Biswas, K. K. (2019). Rapid and sensitive detection of *Citrus tristeza virus* using reverse transcription Loop-mediated Isothermal Amplification (RT-LAMP) assay. *Methods Mol. Biol.* 2015, 143–150.

Gibbs, A., and Mackenzie, A. (1997). A primer pair for amplifying part of the genome of all potyvirids by RT-PCR. *J. Virol. Methods* 63, 9-16.

Gopi, V., Gopal, K., Sankar, T. G., and Palanivel, S. (2010). Detection of citrus yellow mosaic virus by PCR and nucleic acid spot hybridisation using non-radioactive probes in commercial citrus species. *Arch. Phytopathology Plant Protect.* 43, 892-899.

Gosalvez, B., Navarro, J., Lorca, A., Botella, F., Sanchez-Pina, M., and Pallas, V. (2003). Detection of *Melon necrotic spot virus* in water samples and melon plants by molecular methods. *J. Virol. Methods* 113, 87-93.

Ha, C., Coombs, S., Revill, P., Harding, R. M., Vu, M., and Dale, J. L. (2008). Design and application of two novel degenerate primer pairs for the detection and complete genomic characterization of potyviruses. *Arch. Virol.* 153, 25-36.

Hassani-Mehraban, A., Westenberg, M., Verhoeven, J. T. J., van de Vossenberg, B. T. L. H., Kormelink, R., and Roenhorst, J. W. (2016). Generic RT-PCR tests for detection and identification of tospoviruses. *J. Virol. Methods* 233, 89-96.

He, X., Xue, F., Xu, S., and Wang, W. (2016). Rapid and sensitive detection of *Lily symptomless virus* by reverse transcription loop-mediated isothermal amplification. *J. Virol. Methods* 238, 38-41.

Herrera-Vásquez, J., Puchades, A., Elvira-González, L., Jaén-Sanjur, J., Carpino, C., Rubio, L., et al. (2018). Fast detection by loop-mediated isothermal amplification (LAMP) of the three begomovirus species infecting tomato in Panama. *Eur. J. Plant Pathol.* 151, 243-250.

Horns, T., and Jeske, H. (1991). Localization of abutilon mosaic virus (AbMV) DNA within leaf tissue by in situ hybridization. *Virology* 181, 580-588.

Hsu, Y., Yeh, T., and Chang, Y. (2005). A new combination of RT-PCR and reverse dot blot hybridization for rapid detection and identification of potyviruses. *J. Virol. Methods* 128, 54-60.

Huguenot, C., Van Den Dobblesteen, G., De Haan, P., Wagemakers, C., Drost, G., Osterhaus, A., et al. (1990). Detection of tomato spotted wilt virus using monoclonal antibodies and riboprobes. *Arch. Virol.* 110, 47-62.

Jelkmann, W., Maiss, E., Breyel, E., and Casper, R. (1988). Production and use of cDNA clones from arabis mosaic virus. *Ann. Appl. Biol.* 113, 483-491.

Juan, M., Gu, Q., Lin, S., Bin, P., Liu, L., Tian, Y., et al. (2007). Dot-blot hybridization for detection of five cucurbit viruses by digoxigenin-labelled cDNA probes. *Agr. Sci. China* 6, 1450-1455.

Karasev, A. V., Nikolaeva, O. V., Koonin, E. V., Gumpf, D. J., and Garnsey, S. M. (1994). Screening of the closterovirus genome by degenerate primer-mediated polymerase chain reaction. *J. Gen. Virol.* 75, 1415-1422.

Kil, E., Kim, S., Lee, Y., Kang, E., Lee, M., Cho, S., et al. (2015). Advanced loop-mediated isothermal amplification method for sensitive and specific detection of *Tomato chlorosis virus* using an uracil DNA glycosylase to control carry-over contamination. *J. Virol. Methods* 213, 68-74.

Kuan, C., Wu, M., Lu, Y., and Huang, H. (2010). Rapid detection of squash leaf curl virus by loop-mediated isothermal amplification. *J. Virol. Methods* 169, 61-65.

Le, D. T., Netsu, O., Uehara-Ichiki, T., Shimizu, T., Choi, I., Omura, T., et al. (2010). Molecular detection of nine rice viruses by a reverse-transcription loop-mediated isothermal amplification assay. *J. Virol. Methods* 170, 90-93.

Lee, M., Yang, M., Hseu, Y., Lai, G., Chang, W., Hsu, Y., et al. (2011). One-step reverse transcription loop-mediated isothermal amplification assay for rapid detection of *Cymbidium mosaic virus. J. Virol. Methods* 173, 43-48.

Leone, G., Van Schijndel, H., van Genien, B., and Schoen, C. (1997). Direct detection of potato leafroll virus in potato tubers by immunocapture and the isothermal nucleic acid amplification method NASBA. *J. Virol. Methods* 66, 19-27.

Letschert, B., Adam, G., Lesemann, D., Willingmann, P., and Heinze, C. (2002). Detection and differentiation of serologically cross-reacting tobamoviruses of economical importance by RT-PCR and RT-PCR-RFLP. *J. Virol. Methods* 106, 1-10.

Li, Y., Tan, G., Lan, P., Zhang, A., Liu, Y., Li, R., et al. (2018). Detection of tobamoviruses by RT-PCR using a novel pair of degenerate primers. *J. Virol. Methods* 259, 122-128.

Liu, J., Huang, C., Wu, Z., Zhang, X., and Wang, Y. (2010a). Detection of *Tomato aspermy virus* infecting chrysanthemums by LAMP. *Sci. Agric. Sin.* 43, 1288-1294.

Liu, Y., Wang, Z., Qian, Y., Mu, J., Shen, L., Wang, F., et al. (2010b). Rapid detection of tobacco mosaic virus using the reverse transcription loop-mediated isothermal amplification method. *Arch. Virol.* 155, 1681-1685.

Lotos, L., Efthimiou, K., Maliogka, V. I., and Katis, N. I. (2014). Generic detection of poleroviruses using an RT-PCR assay targeting the RdRp coding sequence. *J. Virol. Methods* 198, 1-11.

Maliogka, V., Dovas, C., Efthimiou, K., and Katis, N. (2004). Detection and differentiation of *Comoviridae* species using a semi-nested RT-PCR and a phylogenetic analysis based on the polymerase protein. *J. Phytopathol.* 152, 404-409.

Maliogka, V. I., Dovas, C. I., and Katis, N. I. (2008). Generic and species-specific detection of viruses belonging to an evolutionary distinct lineage within the *Ampelovirus* genus. *J. Virol. Methods* 154, 41-47.

Martín, S., Alioto, D., Milne, R. G., Garnsey, S. M., García, M. L., Grau, O., et al. (2004). Detection of *Citrus psorosis virus* by ELISA, molecular hybridization, RT-PCR and immunosorbent electron microscopy and its association with citrus psorosis disease. *Eur. J. Plant Pathol.* 110, 747-757.

Morozov, S. Y., Ryabov, E., Leiser, R., and Zavriev, S. (1995). Use of highly conserved motifs in plant virus RNA polymerases as the tags for specific detection of carmovirus-related RNA-dependent RNA polymerase genes. *Virology* 207, 312-315.

Mumford, R., Barker, I., and Wood, K. (1996). An improved method for the detection of *Tospoviruses* using the polymerase chain reaction. *J. Virol. Methods* 57, 109-115.

Narváez, G., Skander, B. S., Ayllón, M. A., Rubio, L., Guerri, J., and Moreno, P. (2000). A new procedure to differentiate citrus tristeza virus isolates by hybridisation with digoxigenin-labelled cDNA probes. *J. Virol. Methods* 85, 83–92.

Nie, X. (2005). Reverse transcription loop-mediated isothermal amplification of DNA for detection of *Potato virus Y. Plant Dis.* 89, 605-610.

Nie, X., Bai, Y., Molen, T. A., and Desjardins, D. C. (2008). Development of universal primers for detection of potato carlaviruses by RT-PCR. *J. Virol. Methods* 149, 209-216.

Nikolaeva, O., Yu. Morozov, S., Zakhariev, V., and Skryabin, K. (1990). Improved dot-blot hybridization assay for large-scale detection of potato viruses in crude potato tuber extracts. *J. Phytopathol.* 129, 283-290.

Okuda, M., and Hanada, K. (2001). RT-PCR for detecting five distinct *Tospovirus* species using degenerate primers and dsRNA template. *J. Virol. Methods* 96, 149-156.

Olmos, A., Bertolini, E., and Cambra, M. (2007). Isothermal amplification coupled with rapid flow-through hybridisation for sensitive diagnosis of *Plum pox virus. J. Virol. Methods* 139, 111-115.

Palkovics, L., Burgyan, J., and Balazs, E. (1994). Sensitive non-radioactive nucleic acid hybridization assay for plum pox virus detection. *Res. Virol.* 145, 387-392.

Panno, S., Ferriol, I., Rangel, E. A., Olmos, A., Han, C., Martinelli, F., et al. (2014). Detection and identification of *Fabavirus* species by one-step RT-PCR and multiplex RT-PCR. *J. Virol. Methods* 197, 77-82.

Pappu, S., Brand, R., Pappu, H., Rybicki, E., Gough, K. H., Frenkel, M. J., et al. (1993). A polymerase chain reaction method adapted for selective amplification and cloning of 3' sequences of potyviral genomes: application to dasheen mosaic virus. *J. Virol. Methods* 41, 9-20.

Pappu, H. R., and Druffel, K. L. (2009). Use of conserved genomic regions and degenerate primers in a PCR-based assay for the detection of members of the genus *Caulimovirus*. *J. Virol. Methods* 157, 102-104.

Peng, J., Fan, Z., and Huang, J. (2012a). Rapid detection of *Banana streak virus* by loop-mediated isothermal amplification assay in South China. *J. Phytopathol.* 160, 248-250.

Peng, J., Zhang, J., Xia, Z., Li, Y., Huang, J., and Fan, Z. (2012b). Rapid and sensitive detection of *Banana bunchy top virus* by loop-mediated isothermal amplification. *J. Virol. Methods* 185, 254-258.

Przewodowska, A., Zacharzewska, B., Chołuj, J., and Treder, K. (2015). A one-step, real-time reverse transcription loop-mediated isothermal amplification assay to detect *Potato virus Y. Am. J. Potato Res.* 92, 303-311.

Puchades, A., Carpino, C., Alfaro-Fernández, A., Font-San-Ambrosio, M., Davino, S., Guerri, J., et al. (2017). Detection of *Southern tomato virus* by molecular hybridisation. *Ann. Appl. Biol.* 171, 172-178.

Reddy, R. C., Colvin, J., Muniyappa, V., and Seal, S. (2005). Diversity and distribution of begomoviruses infecting tomato in India. *Arch. Virol.* 150, 845-867.

Robertson, N. L., French, R., and Gray, S. M. (1991). Use of group-specific primers and the polymerase chain reaction for the detection and identification of luteoviruses. *J. Gen. Virol.* 72, 1473-1477.

Rojas, M. (1993). Use of degenerate primers in the polymerase chain reaction to detect whitefly-transmitted geminiviruses. *Plant Dis.* 77, 340-347.

Roy, B., Abou Haidar, M., Sit, T., and Alexander, A. (1988). Construction and use of cloned cDNA biotin and 32P-labeled probes for the detection of papaya mosaic potexvirus RNA in plants. *Phytopathology* 78, 1425-1429.

Rubio, L., Janssen, D., Cuadrado, I. M., Moreno, P., and Guerri, J. (2003). Rapid detection of cucumber vein yellowing virus by tissue-print hybridisation with digoxigenin-labelled cDNA probes. *J. Virol. Methods* 114, 105-107.

Rybicki, E. P., and Hughes, F. L. (1990). Detection and typing of maize streak virus and other distantly related geminiviruses of grasses by polymerase chain reaction amplification of a conserved viral sequence. *J. Gen. Virol.* 71, 2519-2526.

Saade, M., Aparicio, F., Sanchez-Navarro, J., Herranz, M., Myrta, A., Di Terlizzi, B., et al. (2000). Simultaneous detection of the three ilarviruses affecting stone fruit trees by nonisotopic molecular hybridization and multiplex reverse-transcription polymerase chain reaction. *Phytopathology* 90, 1330-1336.

Sabanadzovic, S., Abou-Ghanem, N., Castellano, M., Digiaro, M., and Martelli, G. (2000). Grapevine fleck virus-like viruses in *Vitis. Arch. Virol.* 145, 553-565.

Saldarelli, P., Minafra, A., Martelli, G., and Walter, B. (1994). Detection of grapevine leafroll-associated closterovirus III by molecular hybridization. *Plant Pathol.* 43, 91-96.

Saldarelli, P., Rowhani, A., Routh, G., Minafra, A., and Digiaro, M. (1998). Use of degenerate primers in a RT-PCR assay for the identification and analysis of some filamentous viruses, with special reference to clostero-and vitiviruses of the grapevine. *Eur. J. Plant Pathol.* 104, 945-950.

Sánchez-Navarro, J., Aparicio, F., Rowhani, A., and Pallás, V. (1998). Comparative analysis of ELISA, nonradioactive molecular hybridization and PCR for the detection of prunus necrotic ringspot virus in herbaceous and Prunus hosts. *Plant Pathol.* 47, 780-786.

Sánchez-Navarro, J., Cano, E., and Pallás, V. (1996). Non-radioactive molecular hybridization detection of carnation mottle virus in infected carnations and its comparison to serological and biological techniques. *Plant Pathol.* 45, 375-382.

Sela, I., Reichman, M., and Weissbach, A. (1984). Comparison of dot molecular hybridization and enzyme-linked immunosorbent assay for detecting tobacco mosaic virus in plant tissues and protoplasts. *Phytopathology* 74, 385-389.

Seo, J., Lee, Y., Kim, M., Lee, S., Kim, K., and Choi, H. (2014). A novel set of polyvalent primers that detect members of the genera *Bromovirus* and *Cucumovirus*. *J. Virol. Methods* 203, 112-115.

Stenger, D. C., Mullin, R. H., and Morris, T. J. (1987). Characterization and detection of the strawberry necrotic shock isolate of tobacco streak virus. *Phytopathology* 77, 1330-1337.

Thomson, K., Dietzgen, R., Thomas, J., and Teakle, D. (1996). Detection of pineapple bacilliform virus using the polymerase chain reaction. *Ann. Appl. Biol.* 129, 57-69.

Tian, T., Klaassen, V., Soong, J., Wisler, G., Duffus, J., and Falk, B. (1996). Generation of cDNAs specific to lettuce infectious yellows closterovirus and other whitefly-transmitted viruses by RT-PCR and degenerate oligonucleotide primers corresponding to the closterovirus gene encoding the heat shock protein 70 homolog. *Phytopthology* 86,1167-1173.

Tracy, S. L., Frenkel, M. J., Gough, K. H., Hanna, P., and Shukla, D. D. (1992). Bean yellow mosaic, clover yellow vein, and pea mosaic are distinct potyviruses: evidence from coat protein gene sequences and molecular hybridization involving the 3′ non-coding regions. *Arch. Virol.* 122, 249-261.

Untiveros, M., Perez-Egusquiza, Z., and Clover, G. (2010). PCR assays for the detection of members of the genus *Ilarvirus* and family *Bromoviridae. J. Virol. Methods* 165, 97-104.

Vaira, A., Accotto, G., Costantini, A., and Milne, R. (2003). The partial sequence of RNA 1 of the ophiovirus *Ranunculus white mottle virus* indicates its relationship to rhabdoviruses and provides candidate primers for an ophiovirus-specific RT-PCR test. *Arch. Virol.* 148, 1037-1050.

van der Vlugt, C., Linthorst, H., Asjes, C., Van Schadewijk, A., and Bol, J. (1988). Detection of tobacco rattle virus in different parts of tulip by ELISA and cDNA hybridisation assays. *Eur. J. Plant Pathol.* 94, 149-160.

van der Vlugt, R., Steffens, P., Cuperus, C., Barg, E., Lesemann, D., Bos, L., et al. (1999). Further evidence that shallot yellow stripe virus (SYSV) is a distinct potyvirus and reidentification of Welsh onion yellow stripe virus as a SYSV strain. *Phytopathology* 89, 148-155.

van der Vlugt, René AA, and Berendsen, M. (2002). Development of a general potexvirus detection method. *Eur. J. Plant Pathol.* 108, 367-371.

Varga, A., and James, D. (2006). Use of reverse transcription loop-mediated isothermal amplification for the detection of *Plum pox virus. J. Virol. Methods* 138, 184-190.

Verbeek, M., Tang, J., and Ward, L. I. (2012). Two generic PCR primer sets for the detection of members of the genus *Torradovirus. J. Virol. Methods* 185, 184-188.

Wang, Z., Gu, Q., Sun, H., Li, H., Sun, B., Liang, X., et al. (2014). One-step reverse transcription loop mediated isothermal amplification assay for sensitive and rapid detection of *Cucurbit chlorotic yellows virus. J. Virol. Methods* 195, 63-66.

Wei, Q., Yu, C., Zhang, S., Yang, C., Miriam, K., Zhang, W., et al. (2012). One-step detection of *Bean pod mottle virus* in soybean seeds by the reverse-transcription loop-mediated isothermal amplification. *Virol. J.* 9, 187.

Wetzel, T., Jardak, R., Meunier, L., Ghorbel, A., Reustle, G., and Krczal, G. (2002). Simultaneous RT/PCR detection and differentiation of arabis mosaic and grapevine fanleaf nepoviruses in grapevines with a single pair of primers. *J. Virol. Methods* 101, 63-69.

Willment, J., Martin, D., and Rybicki, E. (2001). Analysis of the diversity of African streak mastreviruses using PCR-generated RFLPs and partial sequence data. *J. Virol. Methods* 93, 75-87.

Wintermantel, W. M., and Hladky, L. L. (2010). Methods for detection and differentiation of existing and new crinivirus species through multiplex and degenerate primer RT-PCR. *J. Virol. Methods* 170, 106-114.

Zhang, Z., Liu, X., Li, D., Yu, J., and Han, C. (2011). Rapid detection of wheat yellow mosaic virus by reverse transcription loop-mediated isothermal amplification. *Virol. J.* 8, 550.

Zhao, K., Liu, Y., and Wang, X. (2010). Reverse transcription loop-mediated isothermal amplification of DNA for detection of Barley yellow dwarf viruses in China. *J. Virol. Methods* 169, 211-214.

Zhao, L., Cheng, J., Hao, X., Tian, X., and Wu, Y. (2012). Rapid detection of tobacco viruses by reverse transcription loop-mediated isothermal amplification. *Arch. Virol.* 157, 2291-2298.

Zheng, L., Rodoni, B., Gibbs, M., and Gibbs, A. (2010). A novel pair of universal primers for the detection of potyviruses. *Plant Pathol.* 59, 211-220.

Zhou, T., Du, L., Fan, Y., and Zhou, Y. (2012). Reverse transcription loop-mediated isothermal amplification of RNA for sensitive and rapid detection of southern rice black-streaked dwarf virus. *J. Virol. Methods* 180, 91-95.

Zong, X., Wang, W., Wei, H., Wang, J., Chen, X., Xu, L., et al. (2014). Rapid detection of *Prunus necrotic ringspot virus* using magnetic nanoparticle-assisted reverse transcription loop-mediated isothermal amplification. *J. Virol. Methods* 208, 85-89.
